# Different Modes of Regulation of the Expression of Dextransucrase in *Leuconostoc lactis* AV1n and *Lactobacillus sakei* MN1

**DOI:** 10.3389/fmicb.2019.00959

**Published:** 2019-05-07

**Authors:** Norhane Besrour-Aouam, Maria Luz Mohedano, Imene Fhoula, Kenza Zarour, Afef Najjari, Rosa Aznar, Alicia Prieto, Hadda-Imene Ouzari, Paloma López

**Affiliations:** ^1^Laboratoire Microorganismes et Biomolécules Actives (LR03ES03), Faculté des Sciences de Tunis, Université Tunis El Manar, Tunis, Tunisia; ^2^Department of Microbial and Plant Biotechnology, Biological Research Center (CIB), Consejo Superior de Investigaciones Científicas (CSIC), Madrid, Spain; ^3^Laboratoire de Microbiologie Appliquée (LMA), Faculté des Sciences de la Nature et de la Vie, Université d’Oran 1 Ahmed Ben Bella, Oran, Algeria; ^4^Department of Microbiology and Ecology, University of Valencia, Burjassot, Spain; ^5^Spanish Type Culture Collection (CECT), University of Valencia, Paterna, Spain; ^6^Department of Preservation and Food Safety Technologies, Institute of Agrochemistry and Food Technology (IATA), Consejo Superior de Investigaciones Científicas (CSIC), Paterna, Spain

**Keywords:** *Leuconostoc lactis*, exopolysaccharides, dextran, lactic acid bacteria, regulation of gene expression

## Abstract

*Leuconostoc lactis* AV1 strain isolated from a Tunisian avocado was characterized as a dextran producer. The promoter P*_dsrLL_* and the *dsrLL* gene encoding the DsrLL dextransucrase responsible for the dextran synthesis were transcriptionally fused to the mCherry coding gene generating the pRCR20 plasmid. Upon plasmid transfer, both AV1n and the dextran non-producing *Leuconostoc mesenteroides* CM70 became red due to expression of the mCherry from the P*_dsrLL_-dsr-mrfp* transcriptional fusion. Characterization of the polymers present in cultures supernatants revealed that the DsrLL encoded from pRCR20 in the recombinant bacteria was able to synthesize dextran. The production of dextran by the DsrLL in AV1n increased in response to low temperature, reaching 10-fold higher levels at 20°C than at 37°C (4.15 g/L versus 0.41 g/L). To analyze if this stress response includes activation at the transcriptional level and if it was only restricted to *Leuconostoc*, AV1n was transformed with plasmids carrying either the P*_dsrLL_*-*mrfp* fusion or the P*_dsrLS_* of *Lactobacillus sakei* MN1 fused to the *mrfp* gene, and the influence of temperature and carbon source on expression from the Dsr promoters was monitored by measurement of the mCherry levels. The overall expression analysis confirmed an induction of expression from P*_dsrLL_* upon growth at low temperature (20°C versus 30°C and 37°C) in the presence of sugars tested (sucrose, glucose, maltose, and fructose). In addition, the presence of sucrose, the substrate of Dsr, also resulted in activation of expression from P*_dsrLL_*. A different behavior was detected, when expression from P*_dsrLS_* was evaluated. Similar levels of fluorescence were observed irrespectively of the carbon source or temperature, besides a sequential decrease at 30°C and 20°C, when sucrose was present in the growth medium. In conclusion, the two types of regulation of expression of Dsr presented here revealed two different mechanisms for environmental adaptation of *Leuconostoc* and *Lactobacillus* that could be exploited for industrial applications.

## Introduction

Lactic acid bacteria (LAB) have been traditionally used for food fermentations as starter or co-adjuvants. This is due to their biosynthetic capabilities and their metabolic pathways, which yield peptides, enzymes, and compounds, involved in quality (e.g., proteases, lipases, and aroma compounds), rheological properties (e.g., exopolysaccharides, EPS) and preservation of the fermented food (e.g., lactic acid, bacteriocins) (Zarour et al., 2017b). In recent years, the increased demand for more “healthy and well-being food” has led to an expanding usage of these microorganisms for the manufacture of functional food products. This is due to the fact that they are able to synthetize several beneficial compounds such as vitamins (e.g., riboflavin and folates), enzymes (e.g., amylases and phytases), or immunomodulatory EPS (Anastasio et al., 2010; Oleksy and Klewicka, 2016; Zarour et al., 2017a; Mosso et al., 2018).

The EPS produced by LAB seem to play a role in their capacity for bacteria-bacteria and eukaryotic cells-bacteria interactions or recognition as well as in the protection of the microbial cells against several environmental and microbial factors (desiccation, pH, osmotic stress, bacteriophage, antibiotics, lysozyme) (Zannini et al., 2016). The total yield of EPS production depends on the producing strain, as well as the growth conditions: (i) medium composition (carbon and nitrogen sources) or (ii) physical factors (temperature, pH, oxygen tension and incubation period) (Patel et al., 2012).

LAB produce a large variety of EPS, which differ in their molecular mass, chemical composition and type of linkage as well as in their degree and type of branching. According to their chemical composition, these biopolymers are classified as: (i) homopolysaccharides (HoPS), composed of only one type of monosaccharide or (ii) heteropolysaccharides (HePS), which contain two or more different types of monosaccharides.

Most of the HoPS contain glucose (glucan), fructose (fructan), or galactose (galactan) residues (Pérez-Ramos et al., 2015). Among them, dextran (a biodegradable α-D-glucan), is the most popular biopolymer used worldwide. Its diversity in degree of branching and chain length confers to it several properties, which defines its application. In general, low-molecular weight dextrans are used in the dermocosmetic industry to promote beneficial cutaneous microbiota and are generally selected for clinical applications (Patel and Goyal, 2011), whereas the high molecular weight versions are mainly used for food products formulation (Kotharia et al., 2014). Thus, dextran is used for making: (i) bakery and confectionary products, to avoid sugar crystallization, to increase moisture retention, and viscosity (reviewed by Pérez-Ramos et al., 2015), and (ii) dairy products, to improve water binding and to enhance the mouthfeel and creaminess of low fat preparations (reviewed by Kotharia et al., 2014). Moreover, the increased consumer demand for gluten-free and low fat food has potentiated the isolation of dextran-producing LAB from various foods (Tieking and Gänzle, 2005; Kaditzky et al., 2008; Bounaix et al., 2010; Nácher-Vázquez et al., 2015; Saravanan and Shetty, 2016; Zarour et al., 2018; Llamas-Arriba et al., 2019) and the study of the capability of LAB for the *in situ* production of the polymer in food matrices such as bread doughs (Galle et al., 2012; Rühmkorf et al., 2012).

There are also evidences supporting that, in addition to food texture improvement, dextran could bring health benefits due to its antiviral and immunomodulatory properties (Nácher-Vázquez et al., 2015; Zarour et al., 2017a). Also, dextran is the major polysaccharide component of kefir grains, and contributes to the symbiotic association of different microrganisms, mainly LAB (including *Lactobacillus and Leuconostoc* species) and yeasts (Gulitz et al., 2011; Fels et al., 2018), and kefir is a traditional fermented milk that due to its beneficial effects for health is considered a functional food (Rosa et al., 2017). Thus, dextran can be used for the future development of new functional fermented food and the state of the art indicates that *in situ* production of the polymer by probiotic-producing LAB could be the best strategy.

Currently, the role of dextran as well as of other HoPS for the producing LAB is not fully understood and it seems to vary between genera, species, and even strains. The use of dextran as a carbon source reserve for the bacteria is not clearly demonstrated, but it has been reported that *Streptococcus mutans* and *Streptococcus sobrinus* can degrade dextran by producing dextranases (Zannini et al., 2016). Analysis of *Lactobacillus reuteri* TMW1.106 mutants deficient in either glucosyltransferase A or inulosucrase indicated that both reuteran (a branched α-D-glucan with both α-1,6- and α-1,4-glycosidic linkages) and inulin (a β-2,6-linked fructan) contribute to bacterial self-aggregations as well as *in vitro* biofilm formation and *in vivo* colonization of mouse gut (Walter et al., 2008). Also, in this animal model, the inulin produced by *Lb. reuteri* 100-23 seems to promote biofilm formation in the murine forestomach (Sims et al., 2011). By contrast, we have previously shown that dextran production by *Lactobacillus sakei* MN1 is concomitant with reduction of the bacterial capacity for self-agglutination, *in vitro* biofilm formation and *in vivo* colonization of zebrafish gut (Nácher-Vázquez et al., 2017a). Moreover, dextran negatively affected binding to Caco-2 epithelial human cells of *Lb. sakei* MN1 and *Leuconostoc mesenteroides* CM9, and did not influence the ability of other *Lc. mesenteroides* strains to adhere to enterocytes (Nácher-Vázquez et al., 2017a; Zarour et al., 2017b). In addition, dextrans seem to play a role in the bacterial response to different stresses. *Lc. mesenteroides* BD3749 produced a high amount of insoluble EPS following exposure to oxidative stress in addition to sucrose. The EPS produced allowed reduction of reactive oxygen species (ROS) accumulated in the cells (Yan et al., 2016). *Leuconostoc gelidium* and *Leuconostoc gasicomitatum* supported strong slime formation after 2–3 weeks storage at 0–6°C (Lyhs et al., 2004). Dextran production was also enhanced by high salinity stress, when *Lactobacillus confusus* TISTR 1498 was grown in submerged fermentation cultivation or in solid state fermentation. This overproduction was unrelated to the biomass yield, which was minimum (Seesuriyachan et al., 2012).

Dextran synthesis involves a single extracellular dextransucrase (Dsr) enzyme encoded by the *dsr* gene. Dextransucrases are members of the GH70 family according to the CAZy classification^1^ and they synthesize dextran by using sucrose as substrate. They catalyze the hydrolysis of the glycosidic bond of sucrose and use the energy released to catalyze the transfer of D-glucopyranosyl residues to the growing polymer, with a concomitant release of fructose (Werning et al., 2012). Furthermore, some Dsr can produce polymers (malto-oligosaccharides) with prebiotic properties using as substrate sucrose and maltose as an acceptor (Dols et al., 1997). Although, numerous studies have been performed for the characterization and industrial application of the Dsr enzymes, little is known about factors affecting the LAB *dsr* gene expression. Nevertheless, transcriptional analysis has demonstrated that during growth of their natural hosts, in the presence of sucrose, an increase of expression of the *Lc. mesenteroides* NRRL B-512F *dsrLM* gene and not of the *Lb. sakei* MN1*dsrLS* gene takes place (Quirasco et al., 1999; Nácher-Vázquez et al., 2017b).

In this work, the EPS produced by *Leuconostoc lactis* AV1n, an avocado isolate, was identified as dextran and regulation of the Dsr genes expression was analyzed by cloning in a plasmid and expressing the Dsr encoding gene, *dsrLL* in *Lc. lactis* and in *Lc. mesenteroides* to prove that it is the enzyme responsible for dextran synthesis. Moreover, comparative transcriptional analysis of the *Lc. lactis* AV1n and *Lb. sakei* MN1 *dsr* genes, in different growth conditions, was carried out to further analyze regulation of Dsr genes expression.

## Materials and Methods

### Bacterial Strains and Growth Conditions

The bacteria used in this work are shown in Table 1. The AV1n strain was isolated from Tunisian avocado using an agar plate containing Man Rogosa Sharpe medium (de Man et al., 1960) supplemented with 2% of sucrose instead of glucose and bromocresol green (25 mg/L) (Dal Bello and Hertel, 2006; Najjari et al., 2008) and after incubation at 30°C during 48 h. This bacterium was classified as a *Leuconostoc lactis* strain, based on the partial sequence of its 16S rRNA gene (GenBank accession number: MK085113). *Leuconostoc* strains were grown in the MRS broth (Pronadisa) supplemented with 2% of either glucose (MRSG), sucrose (MRSS), fructose (MRSF), or maltose (MRSM) and incubated at 20, 30, and 37°C, or in CDM defined medium (Sánchez et al., 2008) supplemented with 0.8% sucrose (CDMS) instead of glucose at 30°C. *Lactococcus lactis* strains were grown in M17 broth (Oxoid) supplemented with 0.5% glucose at 30°C. *Escherichia coli* strains were grown in LB medium containing 10 g/L of tryptone, 5 g/L of yeast extract and 10 g/L of NaCl (pH 7.0) at 37°C. For growth of recombinant bacteria carrying the pRCR promoter probe vector or its derivatives (Table 1), the media were supplemented with chloramphenicol (Cm) at 5 μg/ml for *L. lactis* strains and 10 μg/ml for *Lc. lactis, Lc. Mesenteroides*, and *E. coli* strains.

**Table 1:** Description of bacteria and plasmids used in this work.

Strain or plasmid	Characteristics	References
*Lc. lactis* AV1n	LAB isolated from Tunisian avocado and expressing the DsrLL encoded by the *dsrLL* gene	This study
*Lc. lactis*AV1n[pRCR15]	Strain expressing the P*_dsrLS_*-*mrfp* transcriptional fusion	This study
*Lc. lactis* AV1n[pRCR20]	Strain expressing the P*_dsrLL_*-*dsrLL-mrfp* transcriptional fusion and overproducing DsrLL	This study
*Lc. lactis* AV1n[pRCR21]	Strain expressing the P*_dsrLL_*-*mrfp* transcriptional fusion	This study
*L. lactis* MG1363	Strain derived from *L. lactis* 712 by plasmids curing, used for cloning experiments	Gasson, 1983
*L. lactis* MG1363[pRCR15]	Strain used as source of pRCR15	Nácher-Vázquez et al., 2017b
*L. lactis* MG1363[pRCR20]	Strain used for generation and source of pRCR20	This study
*L. lactis* MG1363[pRCR21]	Strain used for generation and source of pRCR21	This study
*Lc. mesenteroides* CM70	LAB isolated from Algerian camel milk, which carries a *dsrLM* gene encoding a non-functional Dsr	Zarour et al., 2018
*Lc. mesenteroides* CM70[pRCR20]	Strain expressing the P*_dsrLL_*-*dsrLL-mrfp* transcriptional fusion and producing DsrLL	This study
*Lc. mesenteroides* CM70[pRCR21]	Strain carrying the P*_dsrLL_*-*mrfp* transcriptional fusion	This study
*E. coli* DH5α[pRCR]	Strain used as source of pRCR plasmid	Mohedano et al., 2015
pRCR	A promoter probe vector containing the *mrfp* gene, which encodes the fluorescent mCherry protein	Mohedano et al., 2015
pRCR15	This plasmid is a derivative of pRCR carrying the P*_dsrLS_*-*mrfp* transcriptional fusion	This study
pRCR20	This plasmid is a derivative of pRCR carrying the P*_dsrLL_*-*dsrLL-mrfp* transcriptional fusion	This study
pRCR21	This plasmid is a derivative of pRCR carrying the P*_dsrLL_*-*mrfp* transcriptional fusion	This study

### Production, Purification, Quantification, and Characterization of the EPS

For production and analysis of the polymers synthesized by *Leuconostoc* strains, the defined CDM medium supplemented with 0.8% of sucrose was used to avoid any co-precipitation of interfering compounds present in the growth medium during purification of the EPS from culture supernatants. To obtain inocula for the EPS production, strains were grown at 30°C in MRSS to an optical density of 1.0 at a wavelength of 600 nm (OD_600 nm_). Then, after sedimentation by centrifugation (12,000 × *g*, 10 min, 4°C), bacteria were resuspended in the same volume of fresh MRSS, diluted 1:100 in fresh CDMS and the culture incubated at 30°C until the beginning of the stationary phase.

To purify the EPS, bacteria were sedimented by centrifugation (10,651 × *g*, 30 min, 4°C). Then, the EPS present in the supernatants were precipitated by addition of one volume of cold absolute ethanol (v/v), storage at 4°C for 16 h and sedimented by centrifugation (10,651 × *g*, 60 min, 4°C). Afterward, the precipitates were resuspended in water and dialyzed using a membrane with a 12–14 kDa cut-off, against water for 3 days. Finally, the EPS were frozen at -80°C and lyophilized.

The EPS concentration was calculated by measuring the total neutral sugar content by the phenol-sulphuric acid method (Dubois et al., 1956) and using a glucose calibration curve. EPS purity was checked by testing potential contaminants (DNA, RNA, and proteins) using specific fluorescent staining kits and the Qubit^®^ 2.0 fluorometric detection methods (Thermo Fisher Scientific).

EPS characterization was performed basically using the protocols previously described (Notararigo et al., 2013). Briefly, neutral sugars were identified and quantified by gas chromatography (GC), after hydrolysis of the EPS with 3 M trifluoro acetic acid (TFA) for 90 min and derivatization to alditol acetates. The EPS were methylated, hydrolyzed with 3 M TFA for 1 h at 120°C, converted into partially methylated alditol acetates using sodium borodeuteride as the reducing agent and analyzed by GC/mass spectrometry (GC-MS). The linkages present in the EPS were deduced from the mass spectra as well as the retention time of the peaks, and their relative amount from the area of each peak. For Fourier-transformed infrared (IR) spectroscopy (FTIR) analysis, KBr pellets of the samples were first prepared, and the spectra were recorded with a FTIR 4200 instrument (Jasco Corporation) in the range of 4,000–700 cm^-1^. The number of scans per experiment were 50, with a resolution of 4 cm^-1^.

### Genomic and Plasmidic DNA Preparations and DNA Sequencing

For the genomic DNA isolation, bacterial cultures were grown to an OD_600 nm_ of 2.0 at 30°C in MRSG and 1 ml of the culture was sedimented by centrifugation (15,700 × *g*, 5 min, 4°C). Bacteria were washed with phosphate buffered saline (PBS, pH 7.4), resuspended in 100 μl of a solution containing 20% sucrose, 10 mM Tris pH 8.0, 10 mM EDTA, 30 mg/ml of lysozyme, 240 U/ml of mutanolysin, and 80 μg/ml of RNase I, and incubated for 30 min at 37°C. Then, the lysed cells were treated for 2 min with 1% SDS and passed, three times, through a 25 GA needle (0.5 × 16 mm). Afterward, the extracts were deproteinated by treatment (v/v) with a mixture of phenol, chloroform and isoamyl alcohol (50:48:2, v/v/v) for 5 min at 21°C and centrifuged (15,700 × *g*, 10 min, 21°C). The aqueous phases were recovered, and the DNA present in them precipitated with 69.5% (final concentration) ethanol and 83 mM sodium acetate pH 7.0 for 12 h at -20°C. Finally, the precipitates were sedimented by centrifugation (14,500 × *g*, 30 min, -10°C), washed with 1 ml of 70% ethanol, centrifuged (14,500 × *g*, 15 min, -10°C) and finally dissolved in 10 mM Tris buffer pH 8.0.

For the plasmidic DNA isolation, bacteria were grown as above, and samples of the cultures were processed as previously described (Nácher-Vázquez et al., 2017b). Both types of DNA preparations were quantified using the specific fluorescent staining kit and the Qubit^®^ 2.0 fluorometric detection methods (Thermo Fisher Scientific).

DNA sequencing of the *Lc. lactis* AV1n *dsrLL* and the *Lc. mesenteroides* CM70 *dsrLM* genes was performed by the dideoxynucleotide method at Secugen (Madrid, Spain). To characterize the *dsrLL* gene, the dsrLLF and dsrLLR primers (Table 2) were used together with genomic DNA of *Lc. lactis* AV1n and the Phusion High Fidelity Polymerase (Thermo Fisher Scientific) to amplify a 4,900 bp DNA fragment containing the *dsrLL* gene and its putative transcriptional promoter (P*_dsrLL_*). The amplicon was used to determine its nucleotide sequence, together with those and other primers by convergent priming walking. This DNA sequence was deposited in GenBank (accession number MK288168). To characterize the *dsrLM* gene, a DNA fragment encoding a conserved region of the catalytic domain of the LAB Dsr was amplified by using genomic DNA of *Lc. mesenteroides* CM70 and the previously designed dsrF and dsrR primers (Nácher-Vázquez et al., 2017b). Then, the DNA sequence of the amplicon was determined, and a divergent primer walking strategy was followed to obtain the rest of the nucleotide sequence of the *dsrLM* gene and surrounding regions. The 5,498 bp DNA sequence was deposited in the GenBank (accession number MK401907).

**Table 2:** Description of primers used in this work.

Primers	Sequence (5′–3′)	Utilization	Amplicon (bp)
mpRCR	GAAACTCGTGCGTATCCCTC	Primer extension	—
CherryR	CCATTGACCGACCCTTCCAT	Primer extension	—
dsrLLF dsrLLR	TAACATACCGCCCCTAAACTAATTG ATCAAGACATGATGCTGTAAGTCACAC	Cloning of P*_dsrLL_-dsrLL*	4,900
PrdsrLLF PrdsrLLR	TCCCCCCGGGCCGCCCCTAAACTAAT GCTCTAGAGCCCATTAACGATTTGTGT	Cloning of P*_dsrLL_*	318
1F CherryR	ATGCCTCATTATAGCGCTTCC	RT-PCR Amplicon 1	328
2F CherryR	GATACTTCTTAAGTAAATGTTAATCGTTTG	RT-PCR Amplicon 2	243
3F CherryR	CGCTATAACACAAATCGTTAATGGG	RT-PCR Amplicon 3	165

### Construction and Transfer of pRCR Derivatives to LAB Strains

The pRCR promoter probe vector (Mohedano et al., 2015) was used for generation of the recombinant plasmids pRCR20 and pRCR21. The vector carries the *mrfp* gene, which encodes the monomeric mCherry fluorescent protein, without a transcriptional promoter and its preceded by a multicloning site containing among others SmaI and XbaI unique restriction sites.

For construction of pRCR20, the 4,900 bp amplicon containing the P*_dsrLL_* and the *dsrLL* gene (coordinates 1–4,900 in GenBank: MK288168) and described above was blunt end ligated with the T4 DNA ligase (New England Biolabs) to the pRCR vector previously digested with the SmaI restriction enzyme (New England Biolabs).

For construction of pRCR21, a DNA fragment of 318 bp containing the P*_dsrLL_* (coordinates 1–318 in GenBank: MK288168) was amplified using the primers PrdsrLLF and PrdsrLLR (Table 2) in which XmaI or XbaI restriction sites are present, respectively. Then, the amplicon was subjected to digestion with XmaI and XbaI (New England Biolabs) and ligated to pRCR previously digested with the same enzymes.

*L. lactis* MG1363 was independently transformed with each ligation mixture by electroporation (25 μF, 2.5 kV, and 200 Ω in 0.2 cm cuvettes), as previously described (Dornan and Collins, 1987), and transformants were selected in M17G agar plates containing Cm at 5 μg/ml. Then, plasmidic preparations (2 μg) of the recombinant plasmids obtained from either *L. lactis* MG1363[pRCR20] or *L. lactis* MG1363[pRCR21] were used independently to transform *Lc. lactis* AV1n and *Lc. mesenteroides* CM70 by electroporation (25 μF, 2.5 kV, and 200 Ω in 0.4 cm cuvettes) as previously described (David et al., 1989). Transformants were selected in MRSG agar plates containing Cm at 10 μg/ml.

The pRCR15 plasmid was obtained from *L. lactis* MG1363 [pRCR15] and transferred to *Lc. lactis* AV1n by electrotransformation as described above. This recombinant plasmid is a derivative of pRCR, which carries one of the promoter regions of *Lb. sakei* MN1 *dsrLS* gene (designated in this work P*_dsrLS_*) fused to the *mrfp* gene (Nácher-Vázquez et al., 2017b and Table 1).

### Detection of mCherry Fluorescence in LAB Carrying pRCR Plasmid Derivatives

For the analysis of expression from P*_dsrLS_* or P*_dsrLL_, Lc. lactis* AV1n**[**pRCR15] or *Lc. lactis* AV1n**[**pRCR21] were grown in MRSG, MRSS, MRSM, or MRSF at 20, 30, or 37°C until the middle of the exponential phase. Samples of each culture were sedimented by centrifugation (4,500 × *g*, 5 min, 4°C) and washed once with PBS buffer, pH 7.4. Then, samples were concentrated 10-fold in PBS buffer transferred to wells of a 96-well Nunc U96 MicroWell plate (Thermo Fisher Scientific) and maintained at room temperature for 2 h to allow maturation (formation of the chromophore after folding) of the mCherry protein required to become autofluorescent (Supplementary Figure S1) without cell lysis (data not shown). Afterward, the fluorescence levels of the samples were measured in a Varioscan Flash equipment (Thermo Fisher Scientific), using 587 nm and 610 nm wavelengths for excitation and detection of emission, respectively. In addition, appropriate dilutions were prepared to estimate culture biomass by measuring the OD_600 nm._ Three independent trials were performed and the same fresh suspensions, without fixing, were used for phase contrast and fluorescent microscopy analyses with a Leica DM 1000 model microscope (Leica Microsystems) with light source EL6000 and a filter system TX2 ET for red fluorescence detection. The microscope was connected to a DFC3000G camera (Leica microsystems) with CCD sensor. Leica Application suite X Software (Leica Microsystems) was used for image analysis.

*Lc. mesenteroides* CM70[pRCR20] and CM70[pRCR21] were grown in MRSG or MRSS medium at 30°C until the middle of the exponential phase prior to detection of mCherry expression by fluorescence microscopy as detailed above.

### Total RNA Preparations, Primer Extension, and RT-PCR Analyses

For the total RNA isolation, LAB were grown at 20°C (*Lc. lactis* AV1n[pRCR21] and *Lc. lactis* AV1n[pRCR15]) or at 30°C (*Lb. sakei* MN1[pRCR15]) in MRSG medium to an OD_600 nm_ = 1.0. Total RNAs were isolated using the kit “FastRNA pro blue” (QBIOgene) and subjected to electrophoresis in a 0.8% agarose gel at a constant voltage of 100 V for 30 min to check the integrity of the rRNAs. The total RNA concentration was determined using a specific fluorescent staining kit and the Qubit^®^ 2.0 fluorometric detection methods (Thermo Fisher Scientific).

Primer extensions were performed as previously described (Fekete et al., 2003) with some modifications. Two primers complementary to the 5′-region of the *mrfp* gene (Table 2): CherryR (complementary to 140–121 nt in GenBank: KP182347) and mpRCR (complementary to 24–5 nt in GenBank: KP182347) were used. CherryR for analysis of the *dsrLL* transcript from *Lc. lactis* AV1n[pRCR21] and mpRCR for analysis of the *dsrLS* mRNAs from *Lc. lactis* AV1n[pRCR15] and *Lb. sakei* MN1[pRCR15]). The primers were fluorescently labeled with 6-FAM at their 5′-end (Sigma-Aldrich). For the extension reactions, 40 μg of total RNA and 200 pmol of the corresponding primer were mixed and incubated for 5 min at 65°C. Then, the mixture was transferred at 4°C prior to addition of 10 nmol of each dNTP (dATP, dGTP, Dctp, and dTTP) and 200 U of Maxima Reverse Transcriptase (Thermo Fisher Scientific) in the RT buffer (50 mM Tris-HCl pH 8.3, 75 mM KCl, 3 mM MgCl_2_, 10 mM DTT) in a final volume of 50 μl. The reactions were proceeded for 1 h at 50°C, supplemented with 50 μl of TE buffer (10 mM Tris HCl pH 8.0, 1 mM EDTA) to stop them and purified by treatment with a mixture of phenol, chloroform and isoamyl alcohol (50:48:2, v/v/v) for 5 min at room temperature. The cDNA synthesized was then precipitated with 3 volumes of absolute ethanol in the presence of 0.3 mM sodium acetate pH 7.0, stored overnight at -20°C, sedimented by centrifugation (12,000 × *g*, 40 min, -10°C) and resuspended in TE buffer (50 μl). The reaction was further purified using QIAquick Gel extraction kit (QIAGEN), and then analyzed in an Abi 3730 DNA Analyser (Applied Biosystem) capillary electrophoresis instrument using techniques and parameters recommended by the manufacturer. In order to determine the length of the extended products, prior the analysis, the reactions for detection of *dsrLL* (1 μl) or *dsrLM* (4 μl) mRNAs start sites were mixed with DNA sequence reactions of pRCR21 or pRCR15 determined by the dideoxynucleotide method with unlabeled CherryR or mpRCR primers. The Peak Scanner version v1.0 (Applied Biosystems) was used to screen the data and identify major peaks.

The RT-PCR reactions were performed as follows. First, the cDNA synthesis and purification was performed as described above, using a total RNA preparation from *Lc. lactis* AV1n[pRCR21] and the CherryR primer. Then, the PCRs were carried out with the primers 1F, 2F, or 3F (Table 2) and the unlabeled CherryR used as reverse primer. For each reaction an aliquot (2 μl) of the cDNA synthesis reaction mixture was mixed with 0.5 μM (final concentration) of each primer, 200 μM of each of the 4 dNTPs (dATP, dTTP, dCTP, and dGTP) and 1 U of Phusion High Fidelity Polymerase in 1 × Phusion High Fidelity buffer (Thermo Fisher scientific) in a final volume of 50 μl. The annealing temperatures were 60.9 or 63.4°C and the extension times performed at 72°C were 10 s or 13 s for the PCRs performed with 2F-CherryR pair or with either 1F-CherryR and 3F-CherryR pairs. PCRs having as substrate, instead of the synthesized cDNA, either pRCR21 plasmid or the total RNA preparation from *Lc. lactis* AV1n[pRCR21] were performed as control. Each PCR product (2 μl) was loaded in a 1.2% agarose gel and subjected to electrophoresis at a constant voltage of 100 V for 40 min.

### Bioinformatic Analysis

The DNA sequence of *dsrLL* and *dsrLM* genes and their inferred encoded proteins were analyzed with the programs included in the DNASTAR Lasergene 12 (DNAstar Inc.). Homologies of *dsrLL* DNA sequence and its inferred translated products with the NCBI data bases of the National Center for Biotechnology Information (NCBI) were analyzed with the Basic Local Alignment Search Tool (BLAST)^2^. Predictions of the signal peptide cleavage sites in Dsr proteins were performed with SignalP 4.1 program (Petersen et al., 2011)^3^. Prediction of putative specific genetic elements in the DNA sequences was performed with the Genome 2D program^4^.

### Statistical Analysis

Data obtained from three independent experiments were expressed as a mean with their corresponding standard deviation. A two factorial Randomized Complete Block Design (RCBD) was carried out, with experiments, on different days, treated as random blocks. The influence of growth media, temperature, and their join effect on growth rate of the strains and in expression of the *mrfp* transcriptional fusions in the recombinant strains were analyzed with a two-way analysis of variance (ANOVA). A *p* ≤ 0.05 was considered significant. Mean pairwise comparisons were computed with a Tukey’s test (α = 0.05). All analyses were performed with the R software version 3.4.2 (R Core Team, 2017). As the interaction was significant, simple effects were compared. Means with the same letter were not significantly different.

## Results and Discussion

### Characterization of the EPS-Producing *Lc. lactis* AV1n

**FIGURE 1:**
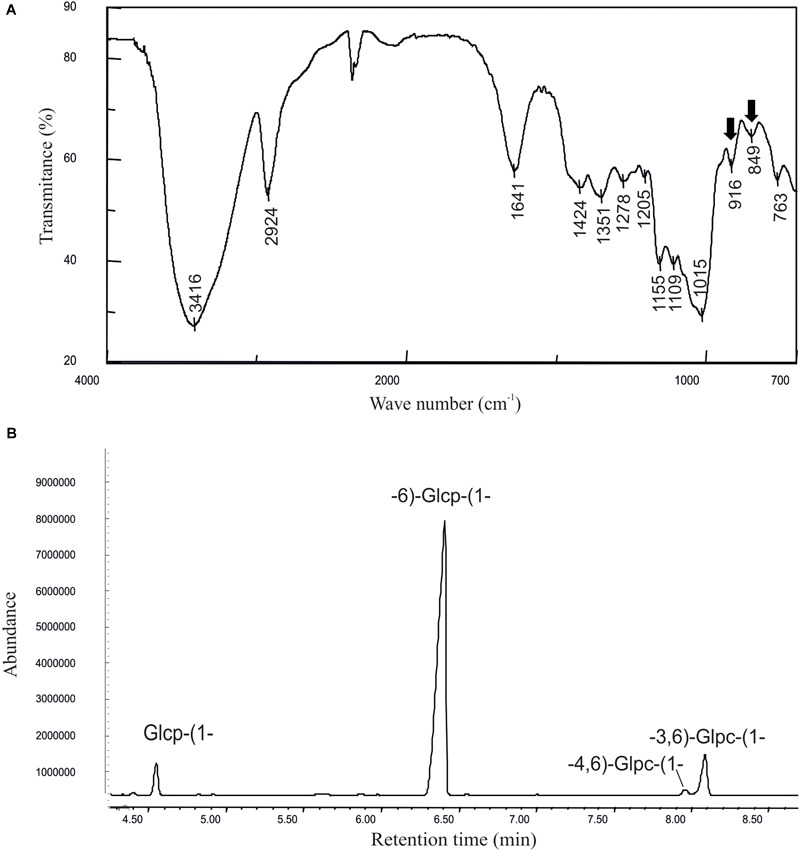
Physicochemical characterization of the dextran produced by *Leuconostoc lactis* AV1n. **(A)** Infrared spectrum and **(B)** methylation analysis of the EPS are depicted.

The current state of the art indicates that future expansion of the functional food products will require new dextran-producing strains for *in situ* production of the polymer. Thus, with the aim to identify new dextran-producing LAB from environments not previously explored and species not previously commercially exploited, the AV1n strain isolated from Tunisian avocado was selected for its ability to generate mucose colonies in a solid MRSS rich medium supplemented with sucrose (Supplementary Figure S2) and classified as belonging to the *Lc. lactis* species by the sequencing of its 16s rRNA coding gene. Then, to determine the levels of EPS production by AV1n, this strain was grown at 30°C in the defined CDM liquid medium supplemented with 0.8% sucrose till the beginning of the stationary phase, and the EPS present in the culture supernatant was quantified by the phenol-sulphuric method. The results showed that *Lc. lactis* AV1n produced 2.25 ± 0.13 g/L, a similar level of EPS to that produced, under the same growth conditions, by the dextran-producing *Lc. mesenteroides* strains (1–3 g/L) (Notararigo et al., 2013; Zarour et al., 2017b) and *Lb. sakei* MN1 (2 g/L) (Nácher-Vázquez et al., 2015). To determine the nature of the polymer produced by AV1n, the EPS was recovered and purified from the culture supernatant by ethanol precipitation and dialysis. Afterward, the EPS characterization was accomplished by: (i) determination of its monomeric composition, (ii) analysis of its monomeric conformation by FTIR and (iii) identification of their linkages by a methylation analysis. The monomeric composition analysis revealed that the polymer was a glucan type HoPS composed only of glucose units (results not shown). The IR spectrum (Figure 1A) was typical of carbohydrates, with two absorption bands around 850 and 920 cm^-1^, characteristic of α-anomers (Notararigo et al., 2013). Methylation analysis (Figure 1B and Table 3) showed that the α-glucan had a main chain of glucopyranose units with α-(1,6) linkages (85%), partially branched at *O*-3 (9%) and at *O*-4 (1.2%) positions with side chains composed of a single α-glucopyranose unit. The overall data defined the polymer as a dextran-type HoPS. Dextrans synthesized by LAB with a similar structure and low proportion of branching (5–10%) at the *O*-3 position is quite common and among others have been previously reported for *Leuconostoc lactis* KC117496 (Saravanan and Shetty, 2016), several *Lc. mesenteroides* strains (Zarour et al., 2017b), and *Lb. sakei* MN1 (Nácher-Vázquez et al., 2015). However, in LAB belonging to the *Leuconostoc* and *Lactobacillus* genera branching at the *O*-4 position is uncommon even in combination with branching at *O*-3 (Fraga Vidal et al., 2011; Llamas-Arriba et al., 2019).

**Table 3:** Methylation analysis of the EPS produced by LAB overproducing DsrLL and their parental strains.

RT (min)	PMAA	Characteristic fragments (m/z)	Deduced linkage type	Proportion of linkage (%)			
					AV1n		CM70
				AV1n	[pRCR20]	CM70	[pRCR20]
4.5	1,4,5-Ac_3_-2,3-Me_2_-Pent	87,102,118,129,189	→ 4)-Pent-(1 → or → 5)-Pent-(1 →	–	–	1.8	–
5.0	1,5-Ac_2_-2,3,4,6-Me_4_-Hex	87, 88, 102, 118, 129, 161, 205	Hexp-(1 →	4.7	6.5	19.1	15.9
6.2	1,2,5-Ac_3_-3,4,6-Me_3_-Hex	87, 88, 101, 129, 130, 161, 190	→ 2)-Hexp-(1 →	–	–	5.3	6.5
6.3	1,3,5-Ac_3_-2,4,6-Me_3_-Glc	101, 118, 129, 161, 234	→ 3)-Glcp-(1 →	–	–	19.9	11.8
6.3	1,4,5-Ac_3_-2,3,6-Me_3_-Gal	88, 102, 118, 130, 162, 233	→ 4)-Galp-(1 → or → 5)-Galf-(1 →	–	–	1.7	0.9
6.4	1,4,5-Ac_3_-2,3,6-Me_3_-Hex	88, 102, 118, 130, 162, 233	→ 4)-Glcp-(1 →	–	–	19.1	-
6.6	1,3,5-Ac_3_-2,4,6-Me_3_-Gal	101, 118, 129, 161, 234	→ 3)-Galp-(1 →	–	–	3.7	2.1
6.7	1,5,6-Ac_3_-2,3,4-Me_3_-Glc	87, 88, 102, 118, 129, 162, 189	→ 6)-Glcp-(1 →	85.0	78.6	17.6	51.6
8.4	1,4,5,6-Ac_4_-2,3-Me_2_-Glc	118, 201, 261	→ 4,6)-Glcp-(1 →	1.2	2.5	0.8	1.1
8.6	1,2,5,6-Ac_4_-3,4-Me_2_-Hex	87, 88, 129, 130, 189, 190	→ 2,6)-Hexp-(1 →	–	–	8.2	1.4
8.6	1,3,5,6-Ac_4_-2,4-Me_2_-Glc	118, 129, 189, 234	→ 3,6)-Glcp-(1 →	9.0	12.3	0.7	5.9
9.2	1,2,5,6-Ac_4_-3,4-Me_2_-Glc	117, 130, 190, 233, 306	→ 2,6)-Galf-(1 →	–	–	2.1	2.0

*There are depicted the retention time (RT) of partially methylated alditol acetate (PMAA) as well as the deduced linkage types and their relative proportions in each strain.*

### Cloning and Plasmid Encoded Expression of the *dsrLL* Gene of *Lc. lactis* AV1n

Dextran synthesis requires only the activity of an extracellular Dsr encoded by a *dsr* gene. Therefore, to identify and further characterize the *dsrLL* gene encoding the DsrLL of *Lc. lactis* AV1n, we took advantage of the fact that the DNA sequence of the *Lc. lactis* Wikim 40 genome was known (GenBank: CP016598.1), and that BLAST analysis against the nucleotide (nr/nt) NCBI database revealed that this LAB carries in its chromosome a *dsr* gene. Thus, we used this gene to design primers and to generate a 4,900 bp amplicon containing the putative *Lc. lactis* AV1n *dsrLL* gene and its flanking regions. Determination of the DNA sequence of the amplicon (GenBank: MK288168) and BLAST analysis revealed the presence of the *dsrLL* gene with an identity of 99 or 97% with its homologs from *Lc. lactis* EG001 (GenBank: GQ213971.1) or from *Leuconostoc garlicum* KFRI01 (GenBank: CP016329.1), *Lc. lactis* WiKim 40 (GenBank: CP016598.1), and *Lc. mesenteroides* LM34 (GenBank: KJ000059.1).

In addition, although the *dsrLS* of *Lb. sakei* MN1 and TMW 1.411 strains have a plasmidic localization (Nácher-Vázquez et al., 2017b; Prechtl et al., 2018a), a large number of the Dsr coding genes have been identified in the chromosomes of *Streptococcus, Leuconostoc*, and *Lactobacillus* strains (Werning et al., 2012). Moreover, BLAST analysis of the 4,900 bp sequenced from the *Lc. lactis* AV1n genome revealed a 97% identity with the *dsr* coding genes and their flanking regions of *Lc. lactis* Wikim 40 and *Lc. garlicum* KFRI01, which are both located on their chromosomes (GenBank CP016598.1 and CP016329.1), suggesting that the sequenced region of AV1n probably has the same location.

**FIGURE 2:**
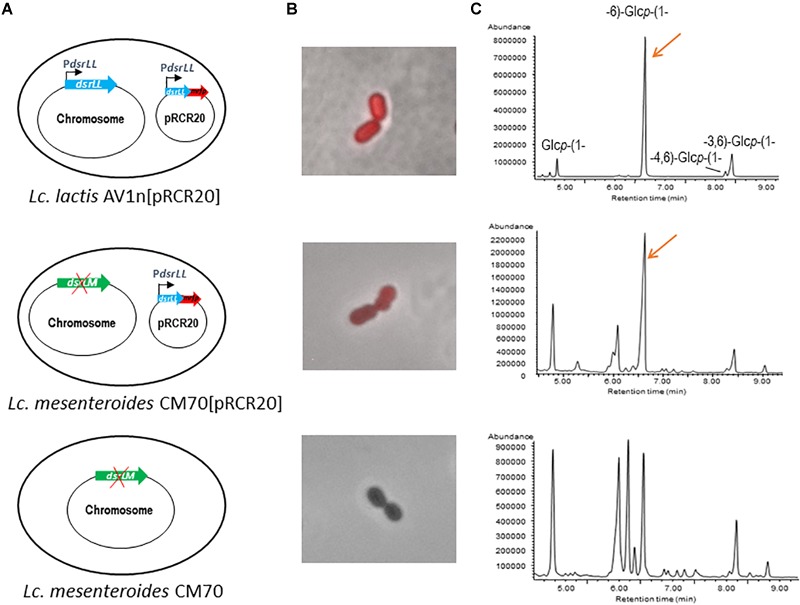
Analysis of influence of over-expression of *dsrLL* gene in *Lc. lactis* AV1n and *Lc. mesenteroides.* **(A)** Diagram of the bacterial genome. **(B)** Overlay of fluorescence and phase contrast images of the analyzed LAB. **(C)** Chromatograms obtained by the methylation analyses of the bacterial EPS.

*In silico* translation of the AV1n *dsrLL* gene of 4,503 nt indicates that it encodes a DsrLL composed of 1,500 amino acids. As the Dsr act extracellularly, these enzymes have to be translocated through the cell membrane thanks to a signal peptide sequence included in the N-terminal region of the protein, which is cleaved off post-translationally, to lead the protein to the secretory pathway (Nielsen et al., 1997). Accordingly, Signal P4.1 program predicted a cleavage site between amino acid residues 41 and 42 of DsrLL characteristic for Gram-positive bacteria. Furthermore, the first few amino acids downstream of the cleavage site of the mature proteins are generally composed of alanine, negatively charged amino acids (D or E) and hydroxy amino acids (S or T) (Nielsen et al., 1997), and in fact DsrLL contains these type of amino acids (D and S). Moreover, BLAST analysis of DsrLL revealed that it carries the Glyco_hydro_70 domain (pfam0234) flanked by the glucan binding repeats and domains characteristics of LAB Dsr (Werning et al., 2012; Nácher-Vázquez et al., 2017b). Furthermore, a LPXTG cell wall anchor domain was detected at the carboxyl-terminal region of the protein. Thus, the overall analysis predicted that the *dsrLL* gene encodes an active DsrLL enzyme bound to the cell wall. Therefore, the enzyme presumably synthesizes a cell surface attached dextran that could protect to the bacteria from extracellular stresses, as it seems to be the case of the EPS synthesizes by the Dsr of *Lb. sakei* TMW1.411 (Prechtl et al., 2018b). Consequently, to test the functionality of DsrLL, the *dsrLL* gene was cloned under the control of its putative promoter in the pRCR promoter probe vector. The resulting plasmid pRCR20 (Figure 2A) carrying the transcriptional fusion P*_dsrLL_-dsrLL*-*mrfp* was stablished in the parental *Lc. lactis* AV1n strain. Transfer of the plasmid showed functionality of P*_dsrLL_* from pRCR20, since conferred to the bacterium a red color (Figure 2B) due to the expression of the mCherry fluorescent protein encoded by the *mrfp* gene. Moreover, it resulted in an increase of the EPS levels in the culture supernatants (from 2.96 ± 0.16 g/L to 4.80 ± 0.25g/L in MRSS at 30°C), indicating an overexpression of a functional DsrLL in AV1n[pRCR20]. Also, methylation analysis of the purified EPS confirmed that the polymers synthesized by the recombinant *Lc. lactis* AV1n[pRCR20] and the parental *Lc. lactis* AV1n strains were essentially the same (Figure 1, 2C and Table 3).

To further test the activity of the DsrLL, the pRCR20 was transferred to *Lc. mesenteroides* CM70. This LAB was previously identified as a dextran-non-producing strain (Zarour et al., 2018). However, in this work a low level of EPS production was observed (0.013 ± 0.002 g/L) and methylation analysis of this polymer revealed that indeed *Lc. mesenteroides* CM70 synthesizes a HePS and not dextran (Figure 2C and Table 3), see details below. Nevertheless, upon transfer of the pRCR20 plasmid, the bacterium became fluorescent (Figure 2B) although with less intensity than that of *Lc. lactis* AV1n[pRCR20] (Figure 2B), and analysis of the EPS present in CM70[pRCR20] culture supernatant showed an increase of the EPS levels to 0.039 ± 0.005 g/L. The carbohydrate content of the polymeric material precipitated from culture supernatants of the *Lc. mesenteroides* parental and recombinant strains were different, amounting to around 50% for CM70[pRCR20] and to only 10% for CM70 (Table 3). In both cases, minor amounts of galactose, mannose and glucose as the main monosaccharides were released upon acid hydrolysis. The molar ratios (Gal:Man:Glc) were (0.7:1:2.4) for the native CM70 and (0.7:1:21.1) in the CM70[pRCR20] (results not shown). Thus, the glucose content of the extracellular material isolated from the recombinant strain was 10-fold higher than in that from the parental natural strain. In addition, the methylation analysis revealed that the dextran produced by AV1[pRCR20] and the EPS from CM70[pRCR20] were similar, with the peak of 1,6-glucopyranose as the main component (Figure 2C and Table 3). The chromatogram of the polymers recovered from CM70 supernatant also displayed this peak, but in a much lower relative proportion. Table 3 gathers the results from the analyses of the extracellular material of the four strains, revealing that AV1n as well as AV1[pRCR20] and CM70[pRCR20] contained the residues characteristic of the 1,3-branched dextran. However, the presence of other components in CM70[pRCR20] indicated that this strain produced, in addition to the dextran, a second EPS. This was more clearly observed in the wild-type CM70 strain, whose analysis disclosed the presence of 1,3-, 1,4-, and 1,6-linked glucopyranose units and 1,2- and 1,2,6-linked hexopyranose residues as the major components of a HePS.

Thus, the overall results revealed that the presence of the *dsrLL* gene in multicopy carried by pRCR20 in the bacterial cytoplasm result in homologous and heterologous production of DsrLL.

Currently, there is limited information available concerning to heterologous expression of Dsr from *Leuconostoc*, and it is mainly based on studies about enzyme activity. The *dexYG* encoding the Dsr of *Lc. mesenteroides* 0326 was heterologously expressed in an active form intracellularly in *E. coli* (Zhang et al., 2008). Also, heterologous expression of *dsrD* from *Lc. mesenteroides* under the control of its natural promoter was accomplished in *L. lactis* MG1363 (Neubauer et al., 2003). In addition, as far as we know, the results presented here, constitute the first report of overexpression of a *dsr* gene of *Lc. lactis* and the first heterologous production of a DsrLL enzyme in *Lc. mesenteroides.* As stated above, this expression did not result in high levels of dextran production in *Lc. mesenteroides* CM70. Also, this behavior has been observed previously, when the *dsrD* gene was heterologous expressed in multicopy, since the DsrD activity was fivefold lower in *L. lactis* than in the parental *Lc. mesenteroides* encoded by a chromosomal gene (Neubauer et al., 2003). Moreover, when *Bacillus megaterium* was used as a host for expression of the *dsrS* gene driven by the inducible xylA promoter, low DsrS activity was obtained and the major limitation for the enzyme production was at the protein folding level (Malten et al., 2005). In the experiments reported here, the conditions for AV1n and CM70 growth were similar, and both strains belong to the same genera. Therefore, the differences in the level of dextran production were probably due to lower activity of the P*_dsrLL_* in *Lc. mesenteroides* as suggested by the low fluorescence of CM70[pRCR20] (Figure 2B) or to an intracellular misfolding of the Dsr. Extracellular protease activity could also be related to the low dextran production in *Lc. mesenteroides*.

Finally, the existence of a *dsrLM* gene in the CM70 strain was investigated to rule out spurious chromosomal implication on the dextran production detected in CM70[pRCR20]. The gene was detected and determination of its nucleotide sequence (1,053–3,962 nt in GenBank MK401907 of 5,498 nt) as well as the flanking regions revealed that this *dsrLM* gene is homologous to the *dsrT* gene of *Lc. mesenteroides* NRRL B-512F (1,054–4,104 nt in GenBank: AB020020.1 of 5,946 nt). In Supplementary Figure S3 is depicted the alignment of both DNA sequences with 97% identity (5,272 nt out of 5,421 nt), with 59 gaps within the 5,421 nt aligned and most of them located at the region homologous to the 5′-end of the *dsrT* gene. As a consequence, the *dsrLM* gene lacks a ribosomal binding site as well as 549 nt of its 5′-region. Consequently, even in the non-probable case that the DsrLM (lacking 183 amino acids of its NH_2_-region) could be synthesized, it will be not active, neither secreted to the environment and consequently could not produce dextran.

### Influence of Temperature and Carbon Source on *Lc. lactis* AV1n Growth and Dextran Production

**FIGURE 3:**
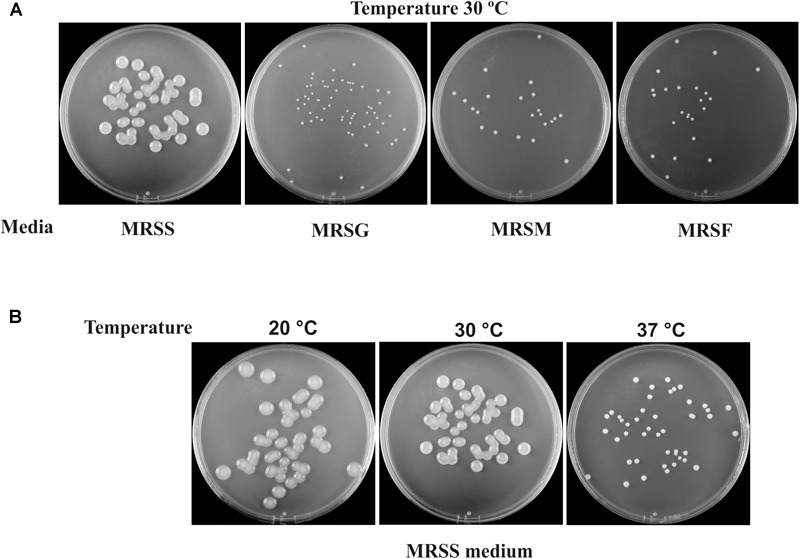
EPS production of *Lc. lactis* AV1n. The images were taken after growth of the bacterium for 3 days in agar plates. **(A)** The growth was performed in the presence of sucrose (MRSS), glucose (MRSG), maltose (MRSM), or fructose (MRSF). **(B)** The growth was performed in MRSS at 20, 30, or 37°C.

**FIGURE 4:**
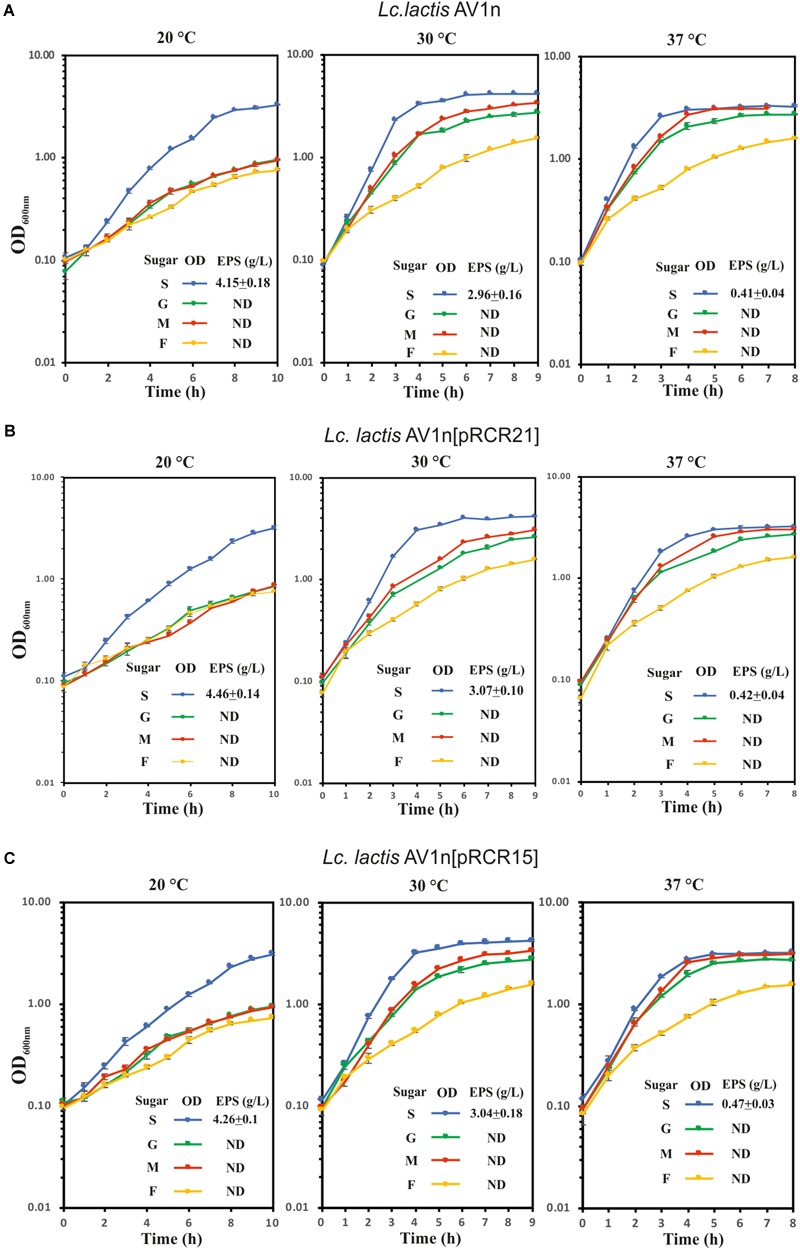
Analysis of growth and dextran production of *Lc. lactis* AV1n and its recombinant derivative strains. **(A)** wild-type strain, **(B)** *Lc. lactis* AV1n[pRCR21], and **(C)** *Lc. lactis* AV1n[pRCR15] grown in MRSG, MRSS, MRSM, or MRSF at 20, 30, and 37°C. Levels of dextran produced by the LAB at A_600 nm_ = 1.0 are also depicted. The mean values and the standard deviation of three independent experiments are depicted.

The dextran-producing *Lc. lactis* AV1n could have interests for its use in functional food development. Therefore, its performance during growth and EPS production in various conditions deserved investigation. Some LAB strain produce more than one EPS and may even produce one HoPS and one HePS (Llamas-Arriba et al., 2019). Thus, growth and production of EPS by *Lc. lactis* AV1n strain in MRS medium containing sucrose, glucose, fructose or maltose at different temperatures were investigated. The bacteria was able to grow at 30°C in MRS containing, sucrose, glucose, maltose or fructose (Figure 3A, 4A), reaching the highest growth rate in MRSS (1.04 ± 0.01) and the lowest in MRSF (0.16 ± 0.10) (Table 4 and Supplementary Figure S4). *Lc. lactis* AV1n was able to produce EPS only in the presence of sucrose. In fact, no slimy colonies were observed when cells were grown at 30°C in MRS-agar supplemented with either glucose, maltose or fructose (Figure 3A), and EPS was only detected upon growth in MRSS liquid medium, when was quantified after precipitation from culture supernatants (Figure 4A). In addition, an influence of the growth temperature was detected on EPS production. Thus, the mucoid aspect of the colonies in MRSS was observed at 20 and 30°C but not at 37°C (Figure 3B). Also, the levels of dextran in MRSS liquid medium increased from 2.96 ± 0.16 g/L to 4.15 ± 0.18 g/L, when AV1n was grown at 20°C instead of at 30°C, and decreased upon growth at 37°C to 0.41 g/L ± 0.04 (Figure 4A). This effect was not due to differences in growth rates, since they were similar at 30 and 37°C (1.04 ± 0.01 g/L and 1.10 ± 0.02 g/L) and drastically lower at 20°C (0.27 ± 0.007 g/L) (Table 4 and Supplementary Figure S3). Moreover, at 20°C in MRSS the final biomass estimated by the OD_600 nm_ = 3.26 was much higher than that in the other media tested OD_600 nm_ = 0.76 or 0.94 in MRSF or in MRSG and MRSM, respectively (Figure 4A).

**Table 4:** Influence of carbon source and temperature in growth rate of *L. lactis* AV1n, *L. lactis* AV1n[pRCR21], and *L. lactis* AV1n[pRCR15].

		Media			
Strains	Temperature	MRSS	MRSG	MRSM	MRSF
AV1n	20°C	0.270 ± 0.007	0.097 ± 0.002	0.097 ± 0.002	0.082 ± 0.003
AV1n	30°C	1.040 ± 0.010	0.331 ± 0.010	0.425 ± 0.010	0.157 ± 0.100
AV1n	37°C	1.100 ± 0.020	0.576 ± 0.006	0.655 ± 0.020	0.195 ± 0.009
AV1[pRCR21]	20°C	0.225 ± 0.007	0.085 ± 0.005	0.075 ± 0.001	0.076 ± 0.003
AV1[pRCR21]	30°C	0.721 ± 0.010	0.261 ± 0.005	0.313 ± 0.100	0.166 ± 0.010
AV1[pRCR21]	37°C	0.795 ± 0.020	0.456 ± 0.010	0.536 ± 0.200	0.205 ± 0.005
AV1[pRCR15]	20°C	0.217 ± 0.010	0,101 ± 0,003	0.092 ± 0.002	0.081 ± 0.003
AV1[pRCR15]	30°C	0.738 ± 0.010	0.256 ± 0.007	0.345 ± 0.008	0.168 ± 0.005
AV1[pRCR15]	37°C	0.778 ± 0.040	0.485 ± 0.010	0.555 ± 0.004	0.208 ± 0.010

### Analysis of *dsrLL* and *dsrLS* Gene Expression

The above results indicated that production of dextran in *Lc. lactis* AV1n increased when temperature decreased. Furthermore, although generally fermentations to produce dextran by *Lc. mesenteroides* are performed at 23–26°C, detailed analysis of the polymer production by *Lc. mesenteroides* under various growth conditions (among others temperature from 20 to 40°C) in batch operation in a bioreactors revealed that optimal activity for Dsr in *Lc. mesenteroides* NRRL B512(f) is 20°C (Santos et al., 2000). Therefore, transcription driven from P*_dsrLL_* could be regulated as a response to environmental temperature. In addition, the expression of the *dsrLL* gene could be induced, when sucrose is present in the growth medium, as it has been previously demonstrated for *Lc. mesenteroides* (Quirasco et al., 1999) or not activated in the presence of this disaccharide as it is the case, among others, for the *dsrLS* gene of *Lb. sakei* MN1 (Nácher-Vázquez et al., 2017b). Moreover, the differences in regulation of gene expression could be due to: (i) the DNA sequence in the promoter regions, (ii) specific transcriptional machinery, or (iii) a host factor of a particular strain or species. Therefore, we envisaged a comparative analysis of expression of *the dsrLL* and the *dsrLS* genes under the control of their natural promoters in *Lc. lactis* AV1n in the presence of different carbon sources or at different temperatures.

**FIGURE 5:**
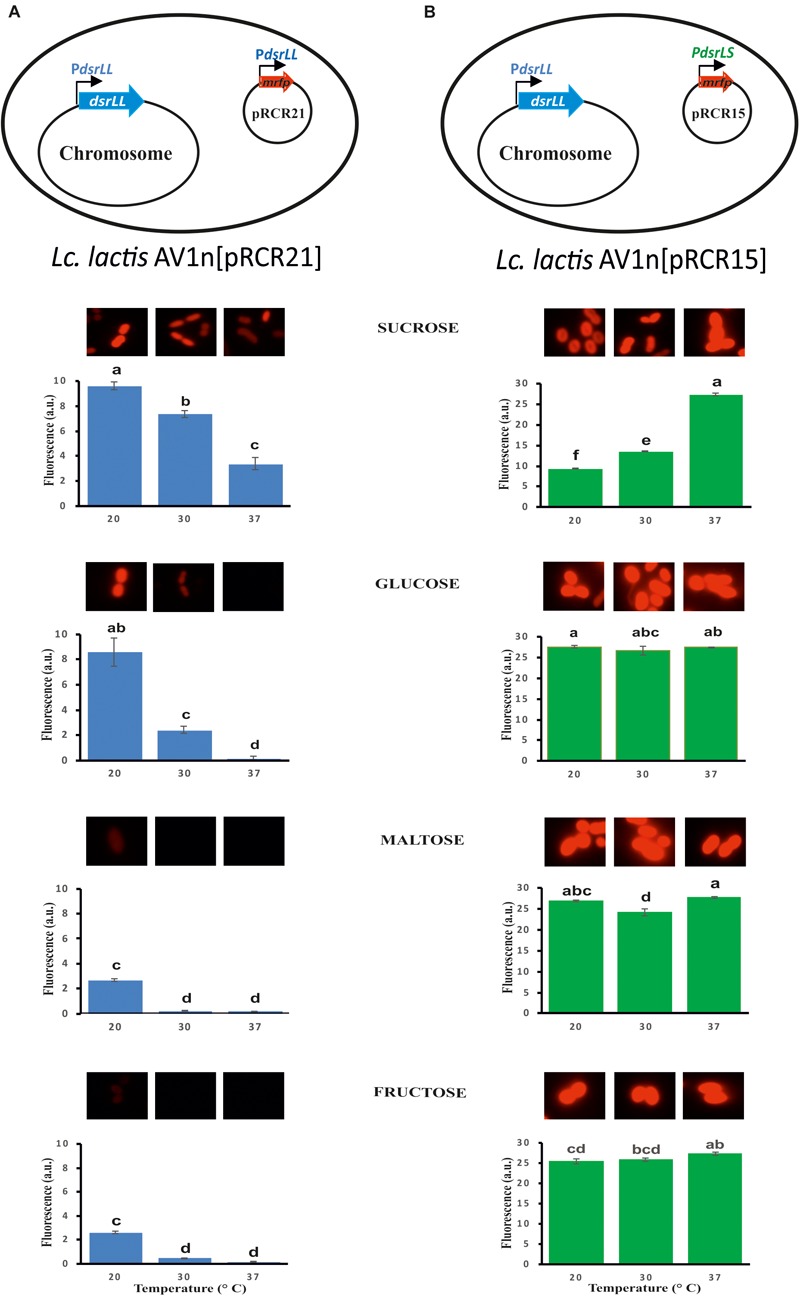
Fluorescent detection of the expression of *mrfp* from the P*_dsrLL_* **(A)** or P*_dsrLS_* **(B)** promoter in the indicated LAB. Bacterial cultures grown in MRSG, MRSS, MRSM and MRSF at 20, 30, and 37°C were analyzed at the middle of the exponential phase by fluorescent microscopy. The specific fluorescence is depicted, and it was calculated as the ratio of the detected fluorescence (5×) and the bacterial biomass estimated from the A_600 nm_ of the culture. The ANOVA statistical analysis of the results is depicted. A *p* ≤ 0.05 was considered significant. Mean pairwise comparisons were computed with a Tukey’s test (α = 0.05). Means with the same letter were not significantly different.

We have previously developed the promoter probe vector pRCR carrying the mCherry protein (*mrfp*) coding gene and deprived of a transcriptional promoter (Mohedano et al., 2015). Moreover, by cloning putative promoter sequences from LAB (*Lactococcus lactis, Lb. sakei*, and *Pediococcus parvulus*), we have validated measurements of the mCherry fluorescence to test regulation of gene expression in the natural or in other heterologous LAB (*L. lactis, Lb. plantarum*, and *Lb. casei*) (Mohedano et al., 2015; Nácher-Vázquez et al., 2017b; Pérez-Ramos et al., 2017) hosts. Thus, pRCR21 carrying the P*_dsrLL_*-*mrfp* transcriptional fusion was constructed and this plasmid as well as pRCR15 carrying the P*_dsrLS_*-*mrfp* transcriptional fusion were transferred to *Lc. lactis* AV1n (Figure 5). Thus, changes in gene expression from the promoters, in the recombinant strains, could be monitored by fluorescent microscopy and spectroscopy detecting the levels of fluorescence emitted by the mCherry protein.

Prior to the gene expression analysis, growth of AV1n, AV1n[pRCR21], and AV1n[pRCR15] exposed to different carbon sources at 20, 30, or 37°C and their EPS production were compared (Figure 4). The ANOVA analysis (Supplementary Figure S4) of the calculated growth rates (Table 4) showed that the interaction medium and temperature was significant, therefore all group means were compared for the interaction. The result revealed that AV1n[pRCR21] and AV1n[pRCR15] showed, in response to shift of media and temperature, a relative growth rate and pattern similar to those of the parental strain in all condition tested, except at 30 and 37°C in MRSS. In these last conditions the growth rate of the recombinant strains was slightly lower than that of AV1n (0.76.1 ± 0.03 versus 1.1 ± 0.04) (Figure 4 and Table 4). In addition, the dextran production in MRSS at different temperatures was similar for the three strains analyzed (Figure 4). These results confirmed that the presence of the plasmids did not alter the general behavior of AV1n and consequently validated the use of the fluorescent labeling to analyze regulation of gene expression.

Therefore, *Lc. lactis* AV1n carrying either pRCR21 or pRCR15 was grown in MRSG, MRSS, MRSM or MRSF at 20, 30, or 37°C until the middle of the exponential phase. Then, optical density of the culture and fluorescence levels were measured and specific fluorescence, referred to the biomass, was calculated and represented (Figure 5). The results revealed that the specific fluorescence level of AV1n[pRCR21] grown in MRSS was the highest at all temperatures tested, e.g., at 30°C were 3.1-, 6.2-, or 48.6-fold higher than that in MRSG, MRSF, or MRSM, respectively. These results support that upon growth in MRSS induction of gene expression from the P*_dsrLL_* promoter takes place. Also, the specific fluorescence level of this strain decreased when the growth temperature was increased. Thus, a change from 20 to 37°C resulted in a decrease of 2.9-, 56-, 14.7-, and 15.2-fold in MRSS, MRSG, MRSM, and MRSF, respectively. These results revealed that the temperature downshift activated transcription from P*_dsrLL_*.

By contrast, for AV1n[pRCR15] no influence of the growth temperature was observed for expression from P*_dsrLS_*, when the bacteria was grown in either MRSG, MRSM or MRSF with an average value of specific fluorescence of 26.5 ± 1.1 (a.u., arbitrary units), equal to that observed in MRSS at 37°C. Therefore, under the previous conditions expression of the *dsrLS* gene behaved as constitutive as it has been described for other LAB (Kralj et al., 2004; Arskold et al., 2007; Bounaix et al., 2010; Gänzle and Follador, 2012). In addition, upon growth in MRSS the specific fluorescence level in AV1n[pRCR15] was affected by the temperature in a fashion opposite to that observed for AV1n[pRCR21], showing a decrease to 13.4 and 9.36 a.u. at 30 and 20°C, respectively. These results correlated with those obtained from our previous transcriptional analysis performed in *Lb. sakei* MN1[pRCR15], which showed that at 30°C sucrose was not an inducer of the *dsrLS* gene expression, and that transcription was higher in MRSG than in MRSS (Nácher-Vázquez et al., 2017b). To explain this phenomenon, we previously suggested that the dextran synthesizes upon growth in MRSS could be the inhibitory agent of its own synthesis by activating a negative feedback mechanism (Nácher-Vázquez et al., 2017b). This previous hypothesis proposes that in the absence or low levels of dextran, there should not be a decrease of expression. Correlating with this hypothesis, in MRSS at 37°C levels of dextran synthesized by *Lc. lactis* AV1n[pRCR15] were low (0.42 g/L) compared with those produced at 30 and 20°C (2.96 and 4.15 g/L), and indeed the inhibitory effect observed by usage of MRSS growth medium on gene expression was not detected at 37°C.

**FIGURE 6:**
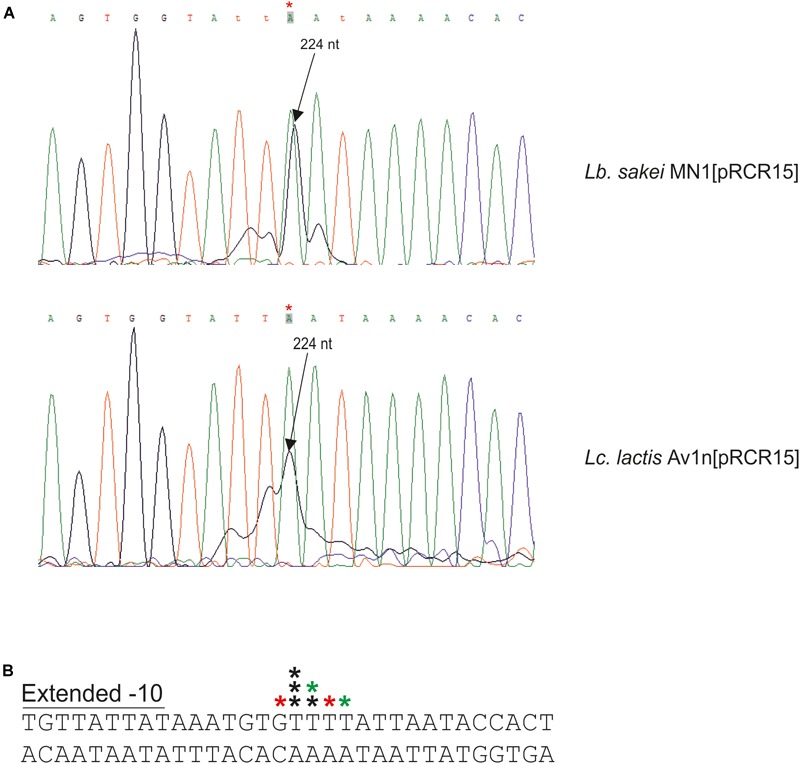
Detection of the start site of the *dsrLS* transcript by primer extension. **(A)** Reactions performed with total RNA of *Lb. sakei* MN1[pRCR15] (1 μl) or *Lc. lactis* AV1n[pRCR15] (4 μl) were analyzed by capillary electrophoresis in conjunction with the DNA sequence of pRCR15 generated with unlabeled mprcr primer. The length of the major extended product (224 nt) is indicated. Due to the 5′-end fluorescent labeling with 6-FAM of the mprcr primer, extended products ran as if they were around 4.5–6.3 nucleotides shorter than the dideoxy sequencing products. In **B** the 5′-ends of the transcript detected in **A** are indicated by stars. The colors of the stars indicate in which primer extension reactions were detected: black, in both; red, in reaction with *Lb. sakei* MN1[pRCR15]; green, in reaction with *Lc. lactis* AV1n [pRCR15]. Also, the DNA region surrounding the mRNA 5′-end and including the -10 region of the *dsrLS* promoter is depicted.

By contrast, Prechtl et al. (2018b) detected an increase of dextran production by *Lb. sakei* TMW 1.411 at low temperature (10°C versus 30°C). In both *Lb. sakei* strains isolated from fermented meat (MN1) or sauerkraut (TMW 1.411), the *dsr* genes are carried by a plasmid, either pMN1 (Nácher-Vázquez et al., 2017b) or p-1.411_1 (Prechtl et al., 2018a), and BLAST analysis revealed a 99% identity of their total nucleotide sequences (data not shown). Moreover, a 100% identity of the region located upstream of their *dsr* genes, which includes the P*_dsrLS_* (Figure 6B), was observed (data not shown). These differences could be due to the fact that P*_dsrLS_* was tested in the *Lc. lactis* heterologous host. However, this was not the case, since higher production of dextran by *Lb. sakei* MN1 at 20°C versus 30°C was not observed (Supplementary Figure S5). In addition, this last strain encodes three plasmids and MN1 only two (pMN1 and pMN2), consequently a transcriptional factor plasmidic-encoded only in TMW 1.411 could play a role in the process. Also, differences in the pMN1 (6.5 ± 1.5 copies per genome equivalent) (Nácher-Vázquez et al., 2017b) and p-1.411_1 (unknown) copy numbers could influence the response to temperature shift. However, this does not seem to be the case, since the replicons of both plasmids are identical (data not shown). Therefore, other post-transcriptional factors involved in generation of an active Dsr or the activity of the enzyme could be responsible for the cold response of TMW 1.411. In fact, translation of the TMW1.411 *dsrLS* gene revealed that DsrLS of TMW1.411 is composed of 1807 amino acids whereas DsrLS MN1 only contains 1767 residues (Supplementary Figure S6). Furthermore, BLAST analysis revealed an identity of 96% between both amino acid sequences and that the extra amino acids of the DsrLS of TMW1.411 are located at the carboxy-terminal region of the enzyme (Supplementary Figure S6). This region, that includes repeated amino acids sequences, seems to be involved in binding of Dsr to the growing chain of the dextran (reviewed in Werning et al., 2012; Nácher-Vázquez et al., 2017b). Therefore, it is feasible that a higher affinity of TMW1.411 protein for the product could result in higher proccesivity and/or turnover of the enzyme at low temperature resulting in a higher production of the EPS.

### Analysis of the *dsrLL* and *dsrLS* Transcripts

To determine the 5′-end of the *dsr* transcripts, primer extensions analyses were performed (Figure 6, 7B). The above and previous RT-PCR results (Nácher-Vázquez et al., 2017b) located P*_dsrLS_* in the insert of pRCR15. Thus, as expected, when total RNA from *Lb. sakei* MN1[pRCR15] and *Lc. lactis* AV1n[pRCR15] were used to generate cDNA complementary to the 5′-end of the *mrfp* mRNA encoded by the pRCR15 plasmid, in both cases a major peak (Figure 6A), was detected corresponding to a T, and upstream an almost canonical extended -10 region (TGNTATtAT) (Figure 6B) was identified, a characteristic binding site of the vegetative σ^70^ factor of the bacterial RNA polymerase without the need of a regulatory protein. Thus, this finding supports the detected constitutive expression from P*dsrLS* in the absence of high levels of dextran production.

**FIGURE 7:**
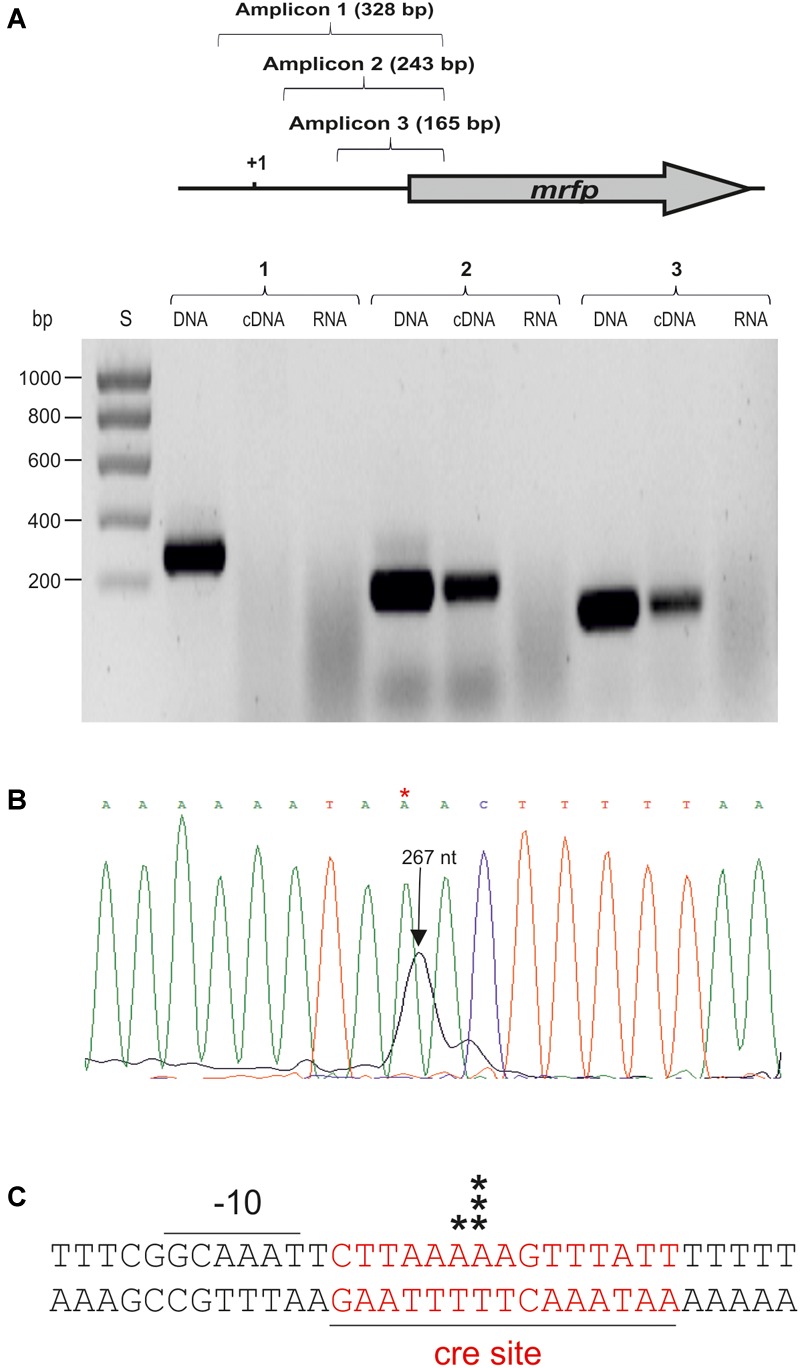
Detection and location of the start site of the *dsrLL* transcript by RT-PCR and primer extension. **(A)** RT-PCR reactions to generate the amplicons 1, 2, or 3 were performed using CherryR as reverse primer and 1F, 2F, or 3F as forward primer. A template was used consisting of the cDNA generated with total RNA of *Lc. lactis* AV1n[pRCR21] and 1F, 2F, or 3F (cDNA); the total RNA (RNA) or the pRCR21 plasmid (DNA). Two microliter of each reaction were analyzed in a 1.2% agarose gel. **(B)** Primer extension reactions performed with total RNA from *Lc. lactis* AV1n[pRCR21] (1 μl) were analyzed by capillary electrophoresis in conjunction with the DNA sequence of pRCR21 generated with the unlabeled CHERRY primer. The length of the major extended product: 267 nt is indicated. In **C** the 5′-ends of the transcript detected in **B** are indicated by black stars. Also, the DNA region surrounding the mRNA start site including the -10 region of the *dsrLL* promoter, and the putative cre site (red letters) is depicted.

The results described in section Analysis of *dsrLL* and *dsrLS* Gene Expression also revealed that the P*_dsrLL_* promoter was included in the insert of the pRCR21 plasmid. Thus, total RNA from AV1n[pRCR21] was used to generate cDNA complementary to the 5′-end of *mrfp* mRNA encoded by the plasmid. A major and a minor peak corresponding to 2 A were detected (Figure 7B). However, inspection of the upstream region did not reveal a canonical -35 or -10 regions characteristic for the σ^70^ promoters, and only a putative gcaAAT degenerated -10 region was observed (Figure 7C). Therefore, to confirm that the location of the start site of the *dsrLL* transcript was not an artifact, RT-PCR reactions were performed for amplification of three regions located upstream of the *mrfp* gene. As expected, the three amplicons were obtained using pRCR21 plasmid as substrate and no amplification was detected, when the substrate was total RNA from AV1n[pRCR21] (Figure 7A). However, when the cDNA complementary to the 5′-end of *mrfp* mRNA, generated as for the primer extension assay, was used as substrate out of three regions tested only the 2nd and 3rd amplicons of 243 and 165 bp were detected (Figure 6A), confirming the location of the 5′-end of the transcript inferred from the primer extension experiment. It should be stated that as far as we know this is the first *dsr* transcript from *Lc. lactis* for which the 5′-end has been determined Consequently, it is not possible to predict how frequent the existence of anomalous promotor sequences in this species might be compared to that detected here. However, it should be stated that non-canonical promoters from *Leuconostoc mesenteroides* has been recently detected by generation of transcriptional fusions with the *cat* gene and testing in *Escherichia coli* (Park et al., 2017). Nevertheless, P*_dsrLL_* does not seem to be very frequent in LAB, since BLAST analysis revealed that the insert cloned in pRCR21, and which includes the promoter region, is not present in the other bacteria carrying homologous *dsr* genes, besides the equivalent regions of *Lc. lactis* Wikim 40 and *Lc. garlicum* KFRI01 genomes.

Concerning to the induction of expression from P*_dsrLL_*, mediated by the presence of sucrose during bacterial growth, prediction of the presence of specific genetic elements with the Genome 2D program revealed a putative catabolic repression element (cre) located downstream of the -10 sequence of P*_dsrLL_* and overlapping the start site of transcription (Figure 7). Its location indicates that binding of the HPr-CcpA complex to the cre would inhibit initiation of transcription of the *dsrLL* gene following a catabolic repression mechanism characteristic of Gram-positive bacteria (Deutscher et al., 1995) in the presence of carbon sources, different from sucrose. Moreover, since a PTS transport system for sucrose is present in *Leuconostoc*, transport of the disaccharide coupled to phosphorylation could affect negatively the levels of HPr-CcpA complex and as a result to induce the expression from P*_dsrLL_*.

With regard to other factors affecting transcription driven from P*_dsrLL_*, it should be stated that transfer of pRCR21 plasmid to *Lc. mesenteroides* CM70 did not confer fluorescence to the recipient strain upon growth in MRSS or MRSG (results not shown) as opposed to pRCR20 transfer. This could be explained if the P*_dsrLL_-mrfp* present in CM70[pRCR21] was not functional. To rule out this possibility, the pRCR21 was extracted from the *Lc. mesenteroides* strain and subsequent sequencing of the pCRC21 DNA insert and the *mrfp* coding gene confirmed the absence of mutations. In addition, when the extracted pRCR21 was transferred to *Lc. lactis* AV1n, the dextran producing bacteria became fluorescent upon growth in MRSS and MRSG. These results demonstrated that the P*_dsrLL_-mrfp* fusion present in pRCR21 was functional and consequently, the lack of fluorescence of CM70[pRCR21] was not due to alterations in the DNA sequence of the plasmid. If we take in consideration that the difference between pRCR21 and pRCR20 plasmids is only that the second encodes DsrLL, and that AV1n and not CM70 background encodes chromosomally DsrLL, it is tempting to suggest that the presence of dextran or the DsrLL enzyme could be related to active expression from P*_dsrLL_*.

Furthermore, in this work a significant activation of transcription driven from P*_dsrLL_* has been detected at low temperature and this could be a response to environmental stress. The study presented here has demonstrated that increase of the *dsrLL* gene transcription as a response to temperature decrease in *Lc. lactis* AV1n correlated with higher production of dextran. This observation cannot be applied to all *Leuconostoc*, as other studies did not detect it (Sarwat et al., 2008), presumably because several post-transcriptional steps, including synthesis, secretion and folding of the Dsr, take place before the dextran is synthesized. Also, it has been reported that maximum Dsr production by *Lactobacillus acidophilus* ST76480.01 was achieved at 30°C and not at 20°C (El-Assad et al., 2013). However, for *Lc. mesenteroides* FT045 B a higher enzyme yield at low temperature (23°C and 25°C) was observed, although the cell growth was low (Cortezi et al., 2005).

In addition, recently, it has been reported that the *dsr* gene expression in *Weissella cibaria* 10 M was induced by cold temperature and not by sucrose (Hu and Gänzle, 2018). Also, although the highest growth and biomass production of this LAB was detected at 30°C, the highest expression of its *dsr* gene was observed at 15°C (Hu and Gänzle, 2018). Correlating with this behavior, the transcription of the *Lc. lactis* AV1n *dsrLL* gene significantly increased at 20°C in all the tested medium, and a significant decrease of the *Lc. lactis* AV1n growth rate was observed when temperature decreased from 30 to 20°C. These results indicate that production of dextran in these two LAB can be included in the response to cold exposure, which provokes decrease of bacterial growth and protein synthesis. The cold general response of the mesophilic bacteria consists in alteration of the membrane composition and a general decrease of the transcription and translation processes, combined with increasing production of specific proteins, among others the cold shock proteins (CSPs) (Barria et al., 2013) which are present in LAB (Kim et al., 1998; Wouters et al., 1999), with the aim to ensure bacterial survival. Therefore, the generation of a biofilm matrix composed of dextran, could constitute a physical barrier protecting cell membrane integrity at low temperature.

The transcriptional signals of the genes encoding CSPs and other cold-inducible genes include a 5′-untranslated region (UTR) of approximately 100 nt between the promoter sequence and the translational start codon (Singh et al., 2014) and in the case of *dsrLL* a spacing of 141 nt exists. In addition, in the UTR there could be the so called cold box (TGAACAACTGC) and dead box (AACAGTGGTA) (Singh et al., 2014), which are not present in the UTR of the *dsrLL* gene. However, in the promoter of the *trmE* gene of *Pseudomonas* species the -10 region sequence is GGAAAT (Singh et al., 2014) almost identical to the GcAAAT of the *dsrLL* gene and that could be related with the activation at low temperature. Nevertheless, further research should be performed to unravel the mechanism involved in this activation in *Lc. lactis.* Moreover, further studies of the mechanism of cold adaptation may lead to the detection of new factors that can selectively modulate the adaptation and growth of LAB under low temperatures, which is important for their applications in the industrial and health fields.

## Conclusion

In the present study, we have shown that *Lc. lactis* AV1n could synthesizes efficiently dextran at low temperature, and consequently could be a good candidate for *in situ* production of dextran in some functional foods (e.g., kefir). Also, in this work by using the pRCR20 plasmid encoding the mCherry protein, it has been proved that DsrLL is the enzyme which synthesizes the dextran in *Lc. lactis*, while analysis of *dsrLL* gene expression allowed for the first time in *Lc. lactis* to detect different dextran production as a response to sucrose and growth temperature. In addition, by using pRCR21 and pRCR15 plasmids, it has been shown that synthesis of Dsr has different regulation mechanisms in *Lc. lactis* and *Lb. sakei*. Therefore, pRCR derivative plasmids expressing mCherry from LAB promoters performed as useful tools to further analyze factors affecting dextran synthesis in order to boost its biotechnological potential for industrial applications.

## Author Contributions

NB-A contributed to all parts of the experimental work and wrote a draft of the manuscript. MM contributed to the design of strategies to develop and analyze the recombinant strains and corrected the manuscript. IF participated in preliminary characterization of the dextran. KZ isolated and contributed to the characterization of the *Lc. mesenteroides* CM70 strain. AN contributed to the study design and to *Lc. lactis AV1n* strain isolation. RA provided the *Lb. sakei MN1* strain and the expertise for analysis of regulation of expression of *dsrLS.* AP designed the experiments and interpreted the results from the physicochemical characterization of the dextran. PL participated in the study conception, data interpretation and generated the final version of the manuscript. H-IO participated in study conception, data interpretation and manuscript revision. All authors have read and approved the final manuscript.

## Conflict of Interest Statement

The authors declare that the research was conducted in the absence of any commercial or financial relationships that could be construed as a potential conflict of interest.

## Footnotes

1 http://www.cazy.org

2 http://blast.ncbi.nlm.nih.gov/Blast.cgi

3 http://www.cbs.dtu.dk/services/SignalP/

4 http://genome2d.molgenrug.nl/

## References

[B1] Anastasio M. Pepe O. Cirillo T. Palomba S. Blaiotta G. Villani F. (2010). Selection and use of phytate-degrading LAB to improve cereal-based products by mineral solubilization during dough fermentation. *J. Food Sci.* 75 M28–M35. 10.1111/j.1750-3841.2009.01402.x 20492182

[B2] Arskold E. Svensson M. Grage H. Roos S. Radstrom P. van Niel E. W. (2007). Environmental influences on exopolysaccharide formation in *Lactobacillus reuteri* ATCC 55730. *Int. J. Food Microbiol.* 116 159–167. 10.1016/j.ijfoodmicro.2006.12.010 17316859

[B3] Barria C. Malecki M. Arraiano C. M. (2013). Bacterial adaptation to cold. *Microbiology* 159(Pt 12) 2437–2443. 10.1099/mic.0.052209-0 24068238

[B4] Bounaix M. S. Robert H. Gabriel V. Morel S. Remaud-Simeon M. Gabriel B. (2010). Characterization of dextran-producing *Weissella* strains isolated from sourdoughs and evidence of constitutive dextransucrase expression. *FEMS Microbiol. Lett.* 311 18–26. 10.1111/j.1574-6968.2010.02067.x 20722740

[B5] Cortezi M. Monti R. Contiero J. (2005). Temperature effect on dextransucrase production by *Leuconostoc mesenteroides* FT 045 B isolated from alcohol and sugar mill plant. *Afr. J. Biotechnol.* 4 279–285.

[B6] Dal Bello F. Hertel C. (2006). Oral cavity as natural reservoir for intestinal *lactobacilli*. *Syst. Appl. Microbiol.* 29 69–76. 10.1016/j.syapm.2005.07.002 16423658

[B7] David S. Simons G. De Vos W. M. (1989). Plasmid transformation by electroporation of *leuconostoc paramesenteroides* and its use in molecular cloning. *Appl. Environ. Microbiol.* 55 1483–1489. 2504108 10.1128/aem.55.6.1483-1489.1989 PMC202890

[B8] de Man J. C. Rogosa M. Sharpe M. E. (1960). A medium for the cultivation of *lactobacilli*. *J. Appl. Microbiol.* 23 130–135. 10.1111/j.1365-2672.1960.tb00188.x

[B9] Deutscher J. Kuster E. Bergstedt U. Charrier V. Hillen W. (1995). Protein kinase-dependent HPr/CcpA interaction links glycolytic activity to carbon catabolite repression in gram-positive bacteria. *Mol. Microbiol.* 15 1049–1053. 7623661 10.1111/j.1365-2958.1995.tb02280.x

[B10] Dols M. Remaud S. M. Willemot R.-M. Vignon M. R. Monsan P. F. (1997). Structural characterization of the maltose acceptor-products synthesized by *Leuconostoc mesenteroides* NRRL B-1299 dextransucrase. *Carbohy. Res.* 305 549–559. 9648272 10.1016/s0008-6215(97)10063-5

[B11] Dornan S. Collins M. (1987). High efficiency electroporation of *Lactococcus lactis* subps. lactis LM0230. *Lett. Appl. Microbiol.* 23 130–135. 10.1111/j.1472-765X.1990.tb01275.x 1367468

[B12] Dubois M. Gilles K. A. Hamilton J. K. Rebers P. A. Smith F. (1956). Colorimetric method for determination of sugars and related substances. *Anal. Chem.* 28 350–356. 10.1021/ac60111a017

[B13] El-Assad S. A. Abo Shall M. El-Borai A. M. Abedin R. M. A. (2013). Optimization and statistical evaluation of medium components affecting dextran and dextransucrase production by *Lactobacillus acidophilus* ST76480.01. *Life Sci. J.* 10 1746–1753.

[B14] Fekete R. Miller M. J. Chattoraj D. K. (2003). Fluorescently labeled oligonucleotides extension, a rapid and quantitative protocol for primer extension. *Biotechniques* 35 90–98. 12866410 10.2144/03351rr01

[B15] Fels L. Jakob F. Vogel R. F. Wefers D. (2018). Structural characterization of the exopolysaccharides from water kefir. *Carbohy. Polym.* 189 296–303. 10.1016/j.carbpol.2018.02.037 29580412

[B16] Fraga Vidal R. Moulis C. Escalier P. Remaud-Simeon M. Monsan P. (2011). Isolation of a gene from *Leuconostoc citreum* B/110-1-2 encoding a novel dextransucrase enzyme. *Curr. Microbiol.* 62 1260–1266. 10.1007/s00284-010-9851-7 21229247

[B17] Galle S. Schwab C. Dal Bello F. Coffey A. Gänzle M. G. Arendt E. K. (2012). Influence of in-situ synthesized exopolysaccharides on the quality of gluten-free sorghum sourdough bread. *Int. J. Food Microbiol.* 155 105–112. 10.1016/j.ijfoodmicro.2012.01.009 22342455

[B18] Gänzle M. G. Follador R. (2012). Metabolism of oligosaccharides and starch in *lactobacilli*: a review. *Front. Microbiol.* 3:340 10.3389/fmicb.2012.00340 PMC3458588 23055996

[B19] Gasson M. J. (1983). Plasmid complements of *Streptococcus lactis* NCDO 712 and other *Lactic streptococci* after protoplast-induced curing. *J. Bacteriol.* 154 1–9. 6403500 10.1128/jb.154.1.1-9.1983 PMC217423

[B20] Gulitz A. Stadie J. Wenning M. Ehrmann M. A. Vogel R. F. (2011). The microbial diversity of water kefir. *Int. J. Food Microbiol.* 151 284–288. 10.1016/j.ijfoodmicro.2011.09.016 22000549

[B21] Hu Y. Gänzle M. G. (2018). Effect of temperature on production of oligosaccharides and dextran by *Weissella cibaria* 10M. *Int. J. Food Microbiol.* 280 27–34. 10.1016/j.ijfoodmicro.2018.05.003 29772465

[B22] Kaditzky S. Behr J. Stocker A. Kaden P. Gänzle M. G. Vogel R. F. (2008). Influence of pH on the formation of glucan by *Lactobacillus reuteri* TMW 1.106 exerting a protective function against extreme pH values. *Food Biotechnol.* 22 398–418.

[B23] Kim W. S. Khunajakr N. Ren J. Dunn N. W. (1998). Conservation of the major cold shock protein in lactic acid bacteria. *Curr. Microbiol.* 37 333–336. 9767713 10.1007/s002849900387

[B24] Kotharia D. Das D. Patel S. Goyala A. (2014). “Dextran and food application,” in *Polysaccharides* eds Ramawat K. G. Mérillon J. M. (Cham: Springer) 1–16.

[B25] Kralj S. van Geel-Schutten G. H. Dondorff M. M. Kirsanovs S. van der Maarel M. J. Dijkhuizen L. (2004). Glucan synthesis in the genus *Lactobacillus*: isolation and characterization of glucansucrase genes, enzymes and glucan products from six different strains. *Microbiology* 150(Pt 11) 3681–3690. 10.1099/mic.0.27321-0 15528655

[B26] Llamas-Arriba M. G. Puertas A. I. Prieto A. López P. Cobos M. Miranda J. I. (2019). Characterization of dextrans produced by *Lactobacillus mali* CUPV271 and *Leuconostoc carnosum* CUPV411. *Food Hydrocoll.* 89 613–622.

[B27] Lyhs U. Koort J. M. Lundstrom H. S. Bjorkroth K. J. (2004). *Leuconostoc gelidum* and *Leuconostoc gasicomitatum* strains dominated the lactic acid bacterium population associated with strong slime formation in an acetic-acid herring preserve. *Int. J. Food Microbiol.* 90 207–218. 14698102 10.1016/s0168-1605(03)00303-9

[B28] Malten M. Hollmann R. Deckwer W. D. Jahn D. (2005). Production and secretion of recombinant *Leuconostoc mesenteroides* dextransucrase DsrS in *Bacillus megaterium*. *Biotechnol. Bioeng.* 89 206–218. 10.1002/bit.20341 15593264

[B29] Mohedano M. L. García-Cayuela T. Pérez-Ramos A. Gaiser R. A. Requena T. López P. (2015). Construction and validation of a mCherry protein vector for promoter analysis in *Lactobacillus acidophilus*. *J. Ind. Microbiol. Biotechnol.* 42 247–253. 10.1007/s10295-014-1567-4 25533634

[B30] Mosso A. L. Jiménez M. E. Vignolo G. LeBlanc J. G. Samman N. C. (2018). Increasing the folate content of tuber based foods using potentially probiotic lactic acid bacteria. *Food Res. Int.* 109 168–174. 10.1016/j.foodres.2018.03.073 29803439

[B31] Nácher-Vázquez M. Ballesteros N. Canales A. Rodriguez Saint-Jean S. Perez-Prieto S. I. Prieto A. (2015). Dextrans produced by lactic acid bacteria exhibit antiviral and immunomodulatory activity against salmonid viruses. *Carbohydr. Polym.* 124 292–301. 10.1016/j.carbpol.2015.02.020 25839823

[B32] Nácher-Vázquez M. Iturria I. Zarour K. Mohedano M. L. Aznar R. Pardo M. A. (2017a). Dextran production by *Lactobacillus sakei* MN1 coincides with reduced autoagglutination, biofilm formation and epithelial cell adhesion. *Carbohydr. Polym.* 168 22–31. 10.1016/j.carbpol.2017.03.024 28457443

[B33] Nácher-Vázquez M. Ruiz-Masó J. A. Mohedano M. L. Del Solar G. Aznar R. López P. (2017b). Dextransucrase expression is concomitant with that of replication and maintenance functions of the pMN1 plasmid in *Lactobacillus sakei* MN1. *Front. Microbiol.* 8:2281. 10.3389/fmicb.2017.02281 29209293 PMC5702455

[B34] Najjari A. Ouzari H. Boudabous A. Zagorec M. (2008). Method for reliable isolation of *Lactobacillus sakei* strains originating from Tunisian seafood and meat products. *Int. J. Food Microbiol.* 121 342–351. 10.1016/j.ijfoodmicro.2007.11.045 18155310

[B35] Neubauer H. Bauche A. Mollet B. (2003). Molecular characterization and expression analysis of the dextransucrase DsrD of *Leuconostoc mesenteroides* Lcc4 in homologous and heterologous *Lactococcus lactis* cultures. *Microbiology* 149(Pt 4) 973–982. 10.1099/mic.0.26029-0 12686639

[B36] Nielsen H. Engelbrecht J. Brunak S. von Heijne G. (1997). Identification of prokaryotic and eukaryotic signal peptides and prediction of their cleavage sites. *Protein Eng.* 10 1–6. 10.1093/protein/10.1.1 9051728

[B37] Notararigo S. Nácher-Vázquez M. Ibarburu I. Werning M. L. Fernández de Palencia P. Dueñas M. T. (2013). Comparative analysis of production and purification of homo- and hetero-polysaccharides produced by lactic acid bacteria. *Carbohyd. Polym.* 93 57–64. 10.1016/j.carbpol.2012.05.016 23465901

[B38] Oleksy M. Klewicka E. (2016). Exopolysaccharides produced by *Lactobacillus* sp.: biosynthesis and applications. *Crit. Rev. Food Sci. Nutr.* 58 450–462. 10.1080/10408398.2016.1187112 27246190

[B39] Park J. Y. Jeong S.-J. Kim J. A. Kim J. H. (2017). Isolation and characterization of some promoter sequences from *Leuconostoc mesenteroides* SY2 isolated from kimchi. *J. Microbiol. Biotechnol.* 27 1586–1592. 10.4014/jmb.1703.03026 28683528

[B40] Patel S. Goyal A. (2011). Functional oligosaccharides: production, properties and applications. *World J. Microbiol. Biotechnol.* 27 1119–1128.

[B41] Patel S. Majumder A. Goyal A. (2012). Potentials of exopolysaccharides from lactic acid bacteria. *Indian J. Microbiol.* 52 3–12. 10.1007/s12088-011-0148-8 23449986 PMC3298600

[B42] Pérez-Ramos A. Nácher-Vázquez M. Notararigo S. López P. Mohedano M. L. (2015). “Current and future applications of bacterial extracellular polysaccharides,” in *Probiotics, Prebiotics and Synbiotics* eds Preedy V. R. Watson R. R. (Oxford: Elsevier).

[B43] Pérez-Ramos A. Werning M. L. Prieto A. Russo P. Spano G. Mohedano M. L. (2017). Characterization of the sorbitol utilization cluster of the probiotic *Pediococcus parvulus* 2.6: genetic, functional and complementation studies in heterologous hosts. *Front. Microbiol.* 8:2393. 10.3389/fmicb.2017.02393 29259592 PMC5723342

[B44] Petersen T. N. Brunak S. von Heijne G. Nielsen H. (2011). SignalP 4.0: discriminating signal peptides from transmembrane regions. *Nat. Methods* 8 785–786. 10.1038/nmeth.1701 21959131

[B45] Prechtl R. M. Janssen D. Behr J. Ludwig C. Kuster B. Vogel R. F. (2018a). Sucrose-induced proteomic response and carbohydrate utilization of *Lactobacillus sakei* TMW 1.411 during dextran formation. *Front. Microbiol.* 9:2796. 10.3389/fmicb.2018.02796 30532743 PMC6265474

[B46] Prechtl R. M. Wefers D. Jakob F. Vogel R. F. (2018b). Cold and salt stress modulate amount, molecular and macromolecular structure of a *Lactobacillus sakei* dextran. *Food Hydrocoll.* 82 73–81. 10.1016/j.foodhyd.2018.04.003

[B47] Quirasco M. Lopez-Munguia A. Remaud-Simeon M. Monsan P. Farres A. (1999). Induction and transcription studies of the dextransucrase gene in *Leuconostoc mesenteroides* NRRL B-512F. *Appl. Environ. Microbiol.* 65 5504–5509. 10584010 10.1128/aem.65.12.5504-5509.1999 PMC91750

[B48] R Core Team (2017). *R: A Language and Environment for Statistical Computing*. Vienna: R Foundation for Statistical Computing. Available at: http://www.R-project.org/

[B49] Rosa D. Dias M. Grześkowiak Ł Reis S. Conceição L. Peluzio M. (2017). Milk kefir: Nutritional, microbiological and health benefits. *Nutr. Res. Rev.* 30 82–96. 10.1017/S0954422416000275 28222814

[B50] Rühmkorf C. Rübsam H. Becker T. Birk C. Voiges K. Mischnick P. (2012). Effect of structurally different microbial homoexopolysaccharides on the quality of gluten-free bread. *Eur. Food Res. Technol.* 235:139 10.1007/s00217-012-1746-3

[B51] Sánchez C. Neves A. R. Cavalheiro J. dos Santos M. M. García-Quintans N. López P. (2008). Contribution of citrate metabolism to the growth of *Lactococcus lactis* CRL264 at low pH. *Appl. Environ. Microbiol.* 74 1136–1144. 10.1128/AEM.01061-07 18156322 PMC2258601

[B52] Santos M. Teixeira J. Rodrigues A. (2000). Production of dextransucrase, dextran and fructose from sucrose using *Leuconostoc mesenteroides* NRRL B512(f). *Biochem. Eng. J.* 4 177–188.

[B53] Saravanan C. Shetty P. K. (2016). Isolation and characterization of exopolysaccharide from *Leuconostoc lactis* KC117496 isolated from idli batter. *Int. J. Biol. Macromol.* 90 100–106. 10.1016/j.ijbiomac.2015.02.007 25687478

[B54] Sarwat F. Ul Qader S. A. Aman A. Ahmed N. (2008). Production & characterization of a unique dextran from an indigenous *Leuconostoc mesenteroides* CMG713. *Int. J. Biol. Sci.* 4 379–386. 18953402 10.7150/ijbs.4.379 PMC2567811

[B55] Seesuriyachan P. Kuntiya A. Hanmoungjai P. Techapun C. Chaiyaso T. Leksawasdi N. (2012). Optimization of exopolysaccharide overproduction by *Lactobacillus confusus* in solid state fermentation under high salinity stress. *Biosci. Biotechnol. Biochem.* 76 912–917. 10.1271/bbb.110905 22738958

[B56] Sims I. M. Frese S. A. Walter J. Loach D. Wilson M. Appleyard K. (2011). Structure and functions of exopolysaccharide produced by gut commensal *Lactobacillus reuteri* 100-23. *ISME J.* 5 1115–1124. 10.1038/ismej.2010.201 21248858 PMC3146279

[B57] Singh A. K. Sad K. Singh S. K. Shivaji S. (2014). Regulation of gene expression at low temperature: role of cold-inducible promoters. *Microbiology* 160(Pt 7) 1291–1296. 10.1099/mic.0.077594-0 24760969

[B58] Tieking M. Gänzle M. G. (2005). Exopolysaccharides from cereal associated lactobacilli. *Trends Food Sci. Technol.* 16:17. 12571016

[B59] Walter J. Schwab C. Schwab Loach D. M. Gänzle M. G. Tannock G. W. (2008). Glucosyltransferase A (GtfA) and inulosucrase (Inu) of *Lactobacillus reuteri* TMW1.106 contribute to cell aggregation, in vitro biofilm formation, and colonization of the mouse gastrointestinal tract. *Microbiology* 154 72–80. 10.1099/mic.0.2007/010637-0 18174127

[B60] Werning M. L. Notararigo S. Nácher M. Fernández de Palencia P. Aznar R. López P. (2012). “Biosynthesis, purification and biotechnological use of exopolysaccharides produced by lactic acid bacteria,” in *Food Additives* ed. El-Samragy Y. (Rijeka: Intech) 83–114.

[B61] Wouters J. A. Jeynov B. Rombouts F. M. de Vos W. M. Kuipers O. P. Abee T. (1999). Analysis of the role of 7 kDa cold-shock proteins of *Lactococcus lactis* MG1363 in cryoprotection. *Microbiology* 145(Pt 11) 3185–3194. 10.1099/00221287-145-11-3185 10589727

[B62] Yan M. Han J. Xu X. Liu L. Gao C. Zheng H. (2016). Gsy, a novel glucansucrase from *Leuconostoc mesenteroides*, mediates the formation of cell aggregates in response to oxidative stress. *Sci. Rep.* 6:38122. 10.1038/srep38122 27924943 PMC5141493

[B63] Zannini E. Waters D. M. Coffey A. Arendt E. K. (2016). Production, properties, and industrial food application of lactic acid bacteria-derived exopolysaccharides. *Appl. Microbiol. Biotechnol.* 100 1121–1135. 10.1007/s00253-015-7172-2 26621802

[B64] Zarour K. Llamas M. G. Prieto A. Ruas-Madiedo P. Dueñas M. T. Fernández de Palencia P. F. (2017a). Rheology and bioactivity of high molecular weight dextrans synthesised by lactic acid bacteria. *Carbohydr. Polym.* 174 646–657. 10.1016/j.carbpol.2017.06.113 28821115

[B65] Zarour K. Vieco N. Pérez-Ramos A. Nácher-Vázquez M. Mohedano M. L. López P. (2017b). “Food ingredients synthesized by lactic acid bacteria,” in *Microbial Production of Ingredients and Additives* eds Grumezescu A. M. Holban A. M. (Amsterdam: ELSEVIER) 89–124.

[B66] Zarour K. Prieto A. Pérez-Ramos A. Kihal M. López P. (2018). Analysis of technological and probiotic properties of Algerian L.mesenteroides strains isolated from dairy and non-dairy products. *J. Funct. Foods* 49 351–361.

[B67] Zhang H. Hu Y. Zhu C. Zhu B. Wang Y. (2008). Cloning, sequencing and expression of a dextransucrase gene (dexYG) from *Leuconostoc mesenteroides*. *Biotechnol. Lett.* 30 1441–1446. 10.1007/s10529-008-9711-8 18414801

