# Supplementary material for: Different Modes of Regulation of the Expression of Dextransucrase in Leuconostoc lactis AV1n and Lactobacillus sakei MN1
Source: Front Microbiol. 2019 May 7;10:959. doi: 10.3389/fmicb.2019.00959 (PMC6513889; doi:10.3389/fmicb.2019.00959)
Supplement: Supplementary file 1 [file Data_Sheet_1.docx]

Supplementary Material

Different modes of regulation of the expression of dextransucrase in *Leuconostoc lactis* AV1n and *Lactobacillus sakei* MN1

**Besrour Aouam Norhane^1^.^2^, Mohedano M.L.^2^, Fhoula Imene^1^, Zarour Kenza^2,3^, Najjari Afef^1^, Aznar Rosa^4,5,6^, Prieto Alicia^2^, Ouzari Hadda-Imene^1^*, López Paloma^2*^**

^1^Laboratoire Microorganismes et Biomolécules Actives (LR03ES03), Faculté des Sciences de Tunis, Université Tunis El Manar, Tunis, Tunisia

^2^Department of Microorganisms and Plant Biotechnology. Biological Research Center (CIB), CSIC, Madrid, Spain

^3^Laboratoire de Microbiologie Appliquée (LMA), Faculté des Sciences de la Nature et de la Vie, Université d’Oran 1 Ahmed Ben Bella, Oran, Algeria

^4^Department of Microbiology and Ecology, University of Valencia, Burjassot, Spain

^5^Spanish Type Culture Collection (CECT), University of Valencia, Paterna, Spain

^6^Department of Preservation and Food Safety Technologies, Institute of Agrochemistry and Food Technology (IATA), CSIC, Paterna, Spain

*** Correspondence:**

Corresponding Author: Paloma López, [plg@cib.csic.es](mailto:plg@cib.csic.es)

Corresponding Author: Ouzari Hadda-Imene, [ouzari.imene@gmail.com](mailto:ouzari.imene@gmail.com)


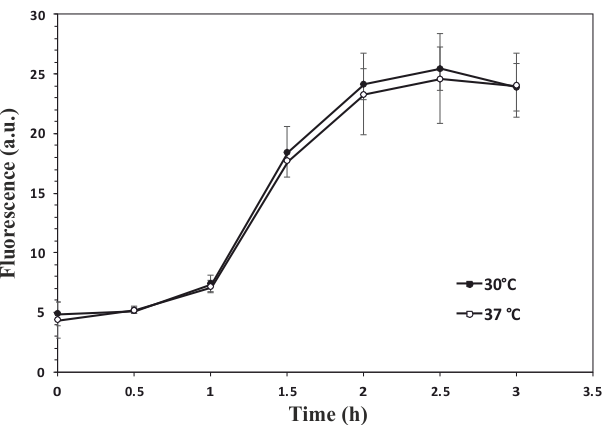


**Supplementary Figure S1. Analysis** of the maturation of the mCherry protein in *Leuconostoc lactis* AV1n [pRCR15] strain. The bacterium was grown in MRSG medium until the middle of the exponential phase and after sedimentation and resuspension in PBS pH 7.4 the fluorescence of the cultures was measured at the indicated times. The depicted values are the average of three independent experiments.


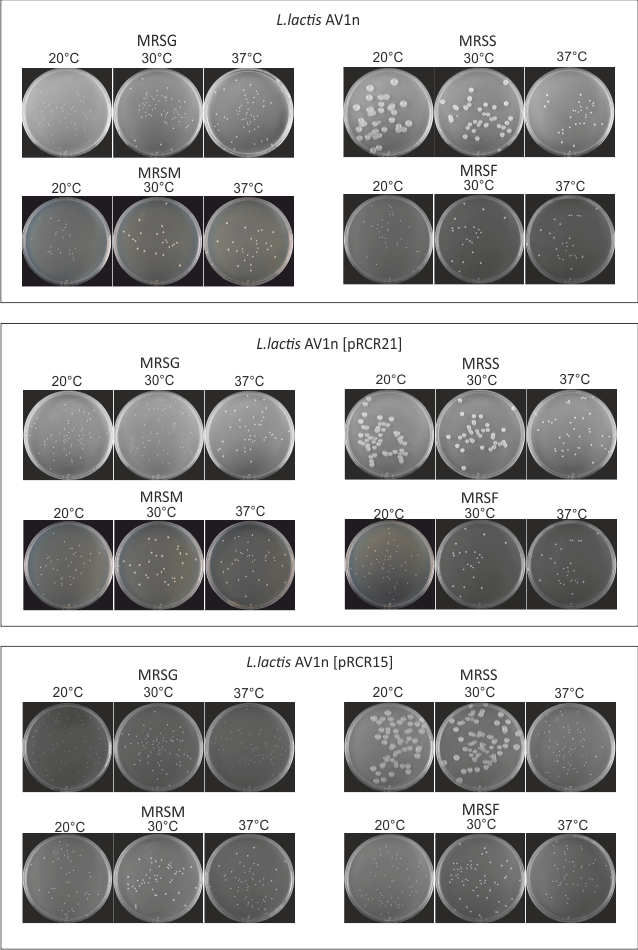


**Supplementary Figure S2**. EPS production by *Leuconostoc lactis* AV1n, *Lc. lactis* AV1n[pRCR21] and *Lc. lactis* AV1n[pRCR15] in solid media. Pictures of the plates were taken after 3 days of growth on MRS agar supplemented with 2% sucrose (MRSS), glucose (MRSG), 2% maltose (MRSM) or 2% fructose (MRSF) at the indicated temperatures.

CM70 1 TTCAACGGATGCCCGTTGACATATTTATCGTAGCGTACTGTTTAAGTATGTCAAATATGT 60

|||||||||||||||||| |||||| |||||||||||||||||||||||||||||||||

NRRL 140 TTCAACGGATGCCCGTTGGCATATTATTCGTAGCGTACTGTTTAAGTATGTCAAATATGT 199

CM70 61 AAGGTGTGATTCTTTCAACATGTATCATGTTAACTACATTCGCCAGTAAACATAAGTCAT 120

||||||||||||||||||||||||||||||||||||||||||||||||||||||||||||

NRRL 200 AAGGTGTGATTCTTTCAACATGTATCATGTTAACTACATTCGCCAGTAAACATAAGTCAT 259

CM70 121 AGAACTGCCAGTAACTAATATTAAAAAAGTAATCAATCAATTCATTCTGATCGTCGATTG 180

||||||||||||||||||||||||||||||||||||||||||||||||||||||||||||

NRRL 260 AGAACTGCCAGTAACTAATATTAAAAAAGTAATCAATCAATTCATTCTGATCGTCGATTG 319

CM70 181 ATGCGACTAGGAAATCTAAGTGATACAAAGTAGACAGCGACATTAAATAATAATGACGCA 240

||||||||||||||||||||||||||||||||||||||||||||||||||||||||||||

NRRL 320 ATGCGACTAGGAAATCTAAGTGATACAAAGTAGACAGCGACATTAAATAATAATGACGCA 379

CM70 241 TGAATGGGTCGCTAGGATTCTTGATTGTATCATTTTGTAATAATTGCGTTAATTGATTCA 300

||||||||||||||||||||||||||||||||||||||||||||||||||||||||||||

NRRL 380 TGAATGGGTCGCTAGGATTCTTGATTGTATCATTTTGTAATAATTGCGTTAATTGATTCA 439

CM70 301 ATTTTTGACGATCATTGTCTTGATAATATTGACTTAAAACAGTTGTCAGGGTTTTTAAGT 360

||||||||||||||||||||||||||||||||||||||||||||||||||||||||||||

NRRL 440 ATTTTTGACGATCATTGTCTTGATAATATTGACTTAAAACAGTTGTCAGGGTTTTTAAGT 499

CM70 361 TGtttttttttttCCAAAAGCAGCCGAAAAATATTCTTCAAATGAAATGTTTAGGGCGTC 420

|| |||||||||||||||||||||||||||||||||||||||||||||||||||||||||

NRRL 500 TG-TTTTTTTTTTCCAAAAGCAGCCGAAAAATATTCTTCAAATGAAATGTTTAGGGCGTC 558

CM70 421 ACACAGTAAAAGTATGGTGTGATCAGTTGGTTCTGTCTTATTGTTCTCTATTCTAGACAC 480

||||||||||||||||||||||||||||||||||||||||||||||||||||||||||||

NRRL 559 ACACAGTAAAAGTATGGTGTGATCAGTTGGTTCTGTCTTATTGTTCTCTATTCTAGACAC 618

CM70 481 CATTGATTGTGATCCAATTAAAACACCCAACCGTTGTTGAGATATTCCTCGTATTTTCCT 540

|||||||||||||||||||||||||| |||||||||||||||||||||||||||||||||

NRRL 619 CATTGATTGTGATCCAATTAAAACACACAACCGTTGTTGAGATATTCCTCGTATTTTCCT 678

CM70 541 AATTTCTTTGATAACAGATCCttttttattattcatatcctcagtaacctataattattt 600

||||||||||||||||||||||||||||||||||||||||||||||||||||||||||||

NRRL 679 AATTTCTTTGATAACAGATCCTTTTTTATTATTCATATCCTCAGTAACCTATAATTATTT 738

CM70 601 aattttttatattttatttaaaagcagtgacttattatatattaaaataaccttaatatt 660

||||||||||||||||||||||||||||||||||||||||||||||||||||||||||||

NRRL 739 AATTTTTTATATTTTATTTAAAAGCAGTGACTTATTATATATTAAAATAACCTTAATATT 798

CM70 661 ttctaattattcaattctgcataatttattttaaatatttgtttcattatatattaaAAT 720

||||||||||||||||||||||||||||||||||||||||||||||||||||||||||||

NRRL 799 TTCTAATTATTCAATTCTGCATAATTTATTTTAAATATTTGTTTCATTATATATTAAAAT 858

CM70 721 AGTTAGTTTTAATGATTAACGTTTGTAAAAAACATTTGTTGACAAAACATTCTTAGTCAT 780

||||||||||||||||||||||||||||||||||||||||||||||||||||||||||||

NRRL 859 AGTTAGTTTTAATGATTAACGTTTGTAAAAAACATTTGTTGACAAAACATTCTTAGTCAT 918

CM70 781 AAATGTTTAGGATCTCACAAGCACGAATAACCGTTTTGATTAGAGAACATAGTAGTTATT 840

|||||||||||||||||||||||| |||||||||||||||||||||| ||||||||||||

NRRL 919 AAATGTTTAGGATCTCACAAGCACAAATAACCGTTTTGATTAGAGAATATAGTAGTTATT 978

CM70 841 TGTGACAAAAAGGGAAAGAATGAAGGGAAGAAGTAAATGAGAAATAGAAATGCAACAAGC 900

|||| |||||||||||||||||||||||||||||||||||||||||||||||||||||||

NRRL 979 TGTGCCAAAAAGGGAAAGAATGAAGGGAAGAAGTAAATGAGAAATAGAAATGCAACAAGC 1038

CM70 901 GTTTTCCGGAAAAAGATGTATAAATCTGGGAAAATGTTAGTCATTGCAGGGAGTGTTTCA 960

||||||||||||||||||||||||||||||||||||||||||||||||||||||||||||

NRRL 1039 GTTTTCCGGAAAAAGATGTATAAATCTGGGAAAATGTTAGTCATTGCAGGGAGTGTTTCA 1098

CM70 961 ATAATTGGTGTTACCAGTTTTATTCAACAAGCAACAAGCTGATGTGT-ATAAATCTGGGA 1019

||||||||||| |||||||||||||||||||| |||||||||||| | | ||| | |

NRRL 1099 ATAATTGGTGTCACCAGTTTTATTCAACAAGC-ACAAGCTGATGTTTCACAAAAC----A 1153

CM70 1020 AAATGTTAGTCATTG-CA-GGGAGTGTTTCAAT--AA-TTGG-TGTTACCA-GT-T--TT 1069

| | |||| | | || || ||| ||||| || |||| || || | || |

NRRL 1154 ATGGGGTAGTAGTGGCCACGGCAGTCGATCAATCGAATTTGGATGCGACTACGTCTGACA 1213

CM70 1070 ATTCAACAAGCAACAAGCTGAT---GTTTC-ACA--A---A-A-CAATGGGGTAGTAGTG 1118

| |||| | |||| || |||| | | | ||| | | | ||| | | || |

NRRL 1214 AATCAATCA-CAAC-AGATGATAAAGCTGCAACAGCAGCTACATCAA-CAGATGATA-AG 1269

CM70 1119 GC--C-ACGGCAGTCGATCAATCGAATTTGGATGCGACTACGTCTG--ACAA-ATCA-AT 1171

|| | || ||| ||| ||| || ||| || | | ||| |||| | || |

NRRL 1270 GCTACAACAACAGCAGATACATC-AA--CAGAT--GATAAAG-CTGCAACAACAGCAGCT 1323

CM70 1172 -CA-CAACAGATGATAAAGCTGCAACAACAGCAGCTACATCAACAGATGATAAAGCTGCA 1229

|| ||||||||||||| ||| ||||||||||||||||||||||||||||||| | ||

NRRL 1324 ACATCAACAGATGATAAGGCTACAACAACAGCAGCTACATCAACAGATGATAA-G--GCT 1380

CM70 1230 ACAACAGCAGCTACATCAACAGATGATAAAGCTGCAACAACAGCAGCTACATCAACAGAT 1289

|||||||||||||||||||||||||||||||||||||||||||||| |||||||||||||

NRRL 1381 ACAACAGCAGCTACATCAACAGATGATAAAGCTGCAACAACAGCAGATACATCAACAGAT 1440

CM70 1290 GATAAGGCTACAACAACAGCAGCTACATCAACAGATGATAAAGCTGCAACAACAGCAGCT 1349

||||||||||||||||||||||||||||||||||||||||| ||| ||||||||||||||

NRRL 1441 GATAAGGCTACAACAACAGCAGCTACATCAACAGATGATAAGGCTACAACAACAGCAGCT 1500

CM70 1350 ACATCAACAGATGATAAAGCTACAACAACAACAGATACATCAACGGATGATAAAGCTGCA 1409

||||||||||||||||| |||||||||||| ||| ||||||||| |||||||||||||||

NRRL 1501 ACATCAACAGATGATAAGGCTACAACAACAGCAGCTACATCAACAGATGATAAAGCTGCA 1560

CM70 1410 ACAACAGCAGCTACATCAACGGATGATAAAACAGCAACAACAGTCGGCACATCTGATAAT 1469

|||||||||| ||||||||||||||||||||||||||||||||||||||||||||||||

NRRL 1561 ACAACAGCAGACACATCAACGGATGATAAAACAGCAACAACAGTCGGCACATCTGATAAT 1620

CM70 1470 AACAATTCAACTACAGCGAGCGATAAAGATGTAAGTTCATCGGCACAAAAAAGTCAAACG 1529

||||||||||||||||||||||||||||||||||||||||||||||||||||||||||||

NRRL 1621 AACAATTCAACTACAGCGAGCGATAAAGATGTAAGTTCATCGGCACAAAAAAGTCAAACG 1680

CM70 1530 ATTGATAACAATTCGAAGACGGCCGATACTACTGCAGCATTAGAAGCTTGTTCAAAGAAC 1589

|||||||||||||||||||||||||||||||||||||||||||||||| ||||||||||

NRRL 1681 ATTGATAACAATTCGAAGACGGCCGATACTACTGCAGCATTAGAAGCTAGTTCAAAGAAT 1740

CM70 1590 CTGAAAACGATTGATGGCAAAACATATTATTACGACGATGATGATCAAGTAAAAAAGAAC 1649

||||||||||||||||||||||||||||||||||||||||||||||||||||||||||||

NRRL 1741 CTGAAAACGATTGATGGCAAAACATATTATTACGACGATGATGATCAAGTAAAAAAGAAC 1800

CM70 1650 TTTGCTACCGTAATTGATGGTAAGGTACTTTATTTTGATAAAGAGACTGGCGCATTAGCT 1709

||||||||||||||||||||||||||||||||||||||||||||||||||||||||||||

NRRL 1801 TTTGCTACCGTAATTGATGGTAAGGTACTTTATTTTGATAAAGAGACTGGCGCATTAGCT 1860

CM70 1710 GATACAAATGACTATCAATTTTTAGAAGGATTGACTAGTGAAAATAATACTTATACGGAG 1769

||||||||||||||||||||||||||||||||||||||||||||||||||||||||||||

NRRL 1861 GATACAAATGACTATCAATTTTTAGAAGGATTGACTAGTGAAAATAATACTTATACGGAG 1920

CM70 1770 CATAATGCCTCAGTTGGTACATCCTCTGATAGTTATACAAACGTTGACGGGTACCTAACA 1829

||||||||||||||||||||| | ||||||||||||||||||||||||||||||||||||

NRRL 1921 CATAATGCCTCAGTTGGTACAACTTCTGATAGTTATACAAACGTTGACGGGTACCTAACA 1980

CM70 1830 GCCGACAGTTGGTACAGGCCTAAGGACATATTAGTCAACGGTCAAAACTGGGAATCATCA 1889

||||||||||||||||||||||||||||||||||||||||||||||||||||||||||||

NRRL 1981 GCCGACAGTTGGTACAGGCCTAAGGACATATTAGTCAACGGTCAAAACTGGGAATCATCA 2040

CM70 1890 AAGGATGACGATTTACGACCATTGTTAATGACTTGGTGGCCAGATAAGGCAACACAAGTA 1949

||||||||||||||||||||||||||||||||||||||||||||||||||||||||||||

NRRL 2041 AAGGATGACGATTTACGACCATTGTTAATGACTTGGTGGCCAGATAAGGCAACACAAGTA 2100

CM70 1950 AACTATTTGAATGCGATGAAGTATTTAGATGCCACTGAAACGGAAACTGTTTATACTTCA 2009

||||||||||||||||||||||||||||||||||||||||||||||||||||||||||||

NRRL 2101 AACTATTTGAATGCGATGAAGTATTTAGATGCCACTGAAACGGAAACTGTTTATACTTCA 2160

CM70 2010 GATGACAGTCAAGACGCTTTGAACAAAGCAGCACAGAACATTCAAGTGAAAATTGAAGAA 2069

||||||||||||||||||||||||||||||||||||||||||||||||||||||||||||

NRRL 2161 GATGACAGTCAAGACGCTTTGAACAAAGCAGCACAGAACATTCAAGTGAAAATTGAAGAA 2220

CM70 2070 AAAATTAGTCAAGAAGGCCAAATACAATGGCTAAAGGATGATATTTCAAAATTTGTTGAT 2129

|||||||||||||||||||||| |||||||||||||||||||||||||||||||||||||

NRRL 2221 AAAATTAGTCAAGAAGGCCAAACACAATGGCTAAAGGATGATATTTCAAAATTTGTTGAT 2280

CM70 2130 AGCCAATCAAATTGGAATATTGCTAGTGAATCAAAAGGAACTGATCATTTGCAAGGTGGT 2189

||||||||||||||||||||||||||||||||||||||||||||||||||||||||||||

NRRL 2281 AGCCAATCAAATTGGAATATTGCTAGTGAATCAAAAGGAACTGATCATTTGCAAGGTGGT 2340

CM70 2190 GCATTGTTGTATGTCAATAGTGATAAAACACCAGATGCCAATTCTGATTATCGATTACTT 2249

||||||||||||||||||||||||||||||||||||||||||||||||||||||||||||

NRRL 2341 GCATTGTTGTATGTCAATAGTGATAAAACACCAGATGCCAATTCTGATTATCGATTACTT 2400

CM70 2250 AATCGCACACCAACAAATCAAACAGGCACGCCTTTGTATACGACAGATCCAACTCAAGGT 2309

||||||||||||||||||||||||||||||||||||||||||||||||||||||||||||

NRRL 2401 AATCGCACACCAACAAATCAAACAGGCACGCCTTTGTATACGACAGATCCAACTCAAGGT 2460

CM70 2310 GGTTATGACTTCCTCTTGGCCAATGATGTGGATAATTCAAACCCAGTTGTTCAAGCAGAA 2369

||||||||||||||||||||||||||||||||||||||||||||||||||||||||||||

NRRL 2461 GGTTATGACTTCCTCTTGGCCAATGATGTGGATAATTCAAACCCAGTTGTTCAAGCAGAA 2520

CM70 2370 CAACTAAATTGGATGTATTACTTGTTAAACTTTGGATCAATTACTAATAACGATGCAGAT 2429

||||||||||||||||||||||||||||||||||||||||||||||||||||||||||||

NRRL 2521 CAACTAAATTGGATGTATTACTTGTTAAACTTTGGATCAATTACTAATAACGATGCAGAT 2580

CM70 2430 GCTAACTTTGATAGTATTCGAGTAGATGCTGTTGATAACGTTGATGCCGACTTATTGCAA 2489

||||||||||||||||||||||||||||||||||||||||||||||||||||||||||||

NRRL 2581 GCTAACTTTGATAGTATTCGAGTAGATGCTGTTGATAACGTTGATGCCGACTTATTGCAA 2640

CM70 2490 ATTGCAGCTGATTATTTCAAGGCAGCATATGGCGTCGATAAGAGTGATGCAATTTCGAAT 2549

|||||||||||||||||||||||||||||||||||||| |||||||||||||||||||||

NRRL 2641 ATTGCAGCTGATTATTTCAAGGCAGCATATGGCGTCGACAAGAGTGATGCAATTTCGAAT 2700

CM70 2550 CAACATGTTTCCATTCTTGAAGACTGGAGTGACAATGATGCTGAATATGTGAAAGACAAT 2609

||||||||||||||||||||||||||||||||||||||||||||||||||||||||||||

NRRL 2701 CAACATGTTTCCATTCTTGAAGACTGGAGTGACAATGATGCTGAATATGTGAAAGACAAT 2760

CM70 2610 GGCGACAATCAATTGTCAATGGATAATAAATTGCGTTTGTCATTAAAATACTCACTCACT 2669

||||||||||||||||||||||||||||||||||||||||||||||||||||||||||||

NRRL 2761 GGCGACAATCAATTGTCAATGGATAATAAATTGCGTTTGTCATTAAAATACTCACTCACT 2820

CM70 2670 ATGCCAGCAGTCGATCAATATGGTAATAAAAGAAGTGGATTAGAACCATTTTTGACAAAT 2729

||||||||||||||||||||||||||||||||||||||||||||||||||||||||||||

NRRL 2821 ATGCCAGCAGTCGATCAATATGGTAATAAAAGAAGTGGATTAGAACCATTTTTGACAAAT 2880

CM70 2730 AGTTTAGTTGATCGTACAAATGATTCGACAGATAATACCGCACAACCCAATTATTCTTTT 2789

||||||||||||||||||||||||||||||||||||||||||||||||||||||||||||

NRRL 2881 AGTTTAGTTGATCGTACAAATGATTCGACAGATAATACCGCACAACCCAATTATTCTTTT 2940

CM70 2790 GTTCGTGCACATGATAGTGAAGTACAAACAGTTATTGCTGAAATTATTAAACAAAGAATT 2849

||||||||||||||||||||||||||||||||||||||||||||||||||||||||||||

NRRL 2941 GTTCGTGCACATGATAGTGAAGTACAAACAGTTATTGCTGAAATTATTAAACAAAGAATT 3000

CM70 2850 GATCCGGATTCTGATGGCTTATCACCAACGATGGACCAATTAACAGAAGCATTTAAAATT 2909

||||||||||||||||||||||||||||||||||||||||||||||||||||||||||||

NRRL 3001 GATCCGGATTCTGATGGCTTATCACCAACGATGGACCAATTAACAGAAGCATTTAAAATT 3060

CM70 2910 TATAATGCTGATCAATTGAAAACAGATAAAGAATTCACACAATTCACACAATATAACATT 2969

||||||||||||||||||||||||||||||| ||||||||||||||||||||

NRRL 3061 TATAATGCTGATCAATTGAAAACAGATAAAG---------AATTCACACAATATAACATT 3111

CM70 2970 CCAAGTACTTATGCCACAATACTAACGAATAAAGATACAGTGCCACGTGTGTACTATGGT 3029

|||||||||||||||||||||||||||||||||||||||||||||||||||||||||||

NRRL 3112 CCAAGTACTTATGCCACAATACTAACGAATAAAGATACAGTGCCACGTGTGTACTATGGG 3171

CM70 3030 GATATGTATACAGATGATGGTCAATACATGGCAACAAAGTCACTTTATTACGATGCAATT 3089

||||||||||||||||||||||||||||||||||||||||||||||||||||||||||||

NRRL 3172 GATATGTATACAGATGATGGTCAATACATGGCAACAAAGTCACTTTATTACGATGCAATT 3231

CM70 3090 GATACTTTGCTGAAGTCTCGTATCAAGTATGTTTCTGGCGGGCAAACAATGTCTATGAAA 3149

||||||||||||||||||||||||||||||||||||||||||||||||||||||||||||

NRRL 3232 GATACTTTGCTGAAGTCTCGTATCAAGTATGTTTCTGGCGGGCAAACAATGTCTATGAAA 3291

CM70 3150 TATATGCAAGGTGATAGTAGTATGGCTGCTGACAGTTATAGAGGCATTTTGACATCAGTT 3209

||||||||||||||||||||||||||||||||||||||||||||||||||||||||||||

NRRL 3292 TATATGCAAGGTGATAGTAGTATGGCTGCTGACAGTTATAGAGGCATTTTGACATCAGTT 3351

CM70 3210 CGTTATGGTAATGGTGCCATGACTGCTACCGATGCAGGGACAAATGAAACACGTACGCAA 3269

||||||||||||||||||||||||||||||||||||||||||||||||||||||||||||

NRRL 3352 CGTTATGGTAATGGTGCCATGACTGCTACCGATGCAGGGACAAATGAAACACGTACGCAA 3411

CM70 3270 GGTATTGCAGTAATTGAAAGTAATAACCCAGATTTGAAGTTGAGCAGTACAGATCAAGTA 3329

||||||||||||||||||||||||||||||||||||||||||||||||||||||||||||

NRRL 3412 GGTATTGCAGTAATTGAAAGTAATAACCCAGATTTGAAGTTGAGCAGTACAGATCAAGTA 3471

CM70 3330 GTTGTAGATATGGGCATAGCGCACAAAAATCAGGCTTATCGTCCTGCTTTGTTAACAACT 3389

||||||||||||||||||||||||||||||||||||||||||||||||||||||||||||

NRRL 3472 GTTGTAGATATGGGCATAGCGCACAAAAATCAGGCTTATCGTCCTGCTTTGTTAACAACT 3531

CM70 3390 AAAGATGGCATAGATACTTATGTATCTGATAGTGATGTCTCACAAAGCTTAATAAGATAT 3449

||||||||||||||||||||||||||||||||||||||||||||||||||||||||||||

NRRL 3532 AAAGATGGCATAGATACTTATGTATCTGATAGTGATGTCTCACAAAGCTTAATAAGATAT 3591

CM70 3450 ACAAATAGTAATGGGCAACTTATTTTCAATAGTTCAGATATTGTTGGTACAGCAAATCCA 3509

||||||||||||||||||||||||||||||||||||||||||||||||||||||||||||

NRRL 3592 ACAAATAGTAATGGGCAACTTATTTTCAATAGTTCAGATATTGTTGGTACAGCAAATCCA 3651

CM70 3510 CAAGTTTCTGGATACTTGGCGGTCTGGGTACCCGTTGGTGCTTCAGATACTCAAGATGCG 3569

||||||||||||||||||||||||||||||||||||||||||||||||||||||||||||

NRRL 3652 CAAGTTTCTGGATACTTGGCGGTCTGGGTACCCGTTGGTGCTTCAGATACTCAAGATGCG 3711

CM70 3570 CGAACTGAAAGTAGTACAGCAACAACTACTGATGGACAAACATTACATTCAAATGCCGCA 3629

||||||||||||||||||||||||||||||||||||||||||||||||||||||||||||

NRRL 3712 CGAACTGAAAGTAGTACAGCAACAACTACTGATGGACAAACATTACATTCAAATGCCGCA 3771

CM70 3630 CTTGATTCTCAAGTTATTTATGAAAGTTTCTCTAACTTCCAATCTACACCAACAACAGAA 3689

||||||||||||||||||||||||||||||||||||||||||||||||||||||||||||

NRRL 3772 CTTGATTCTCAAGTTATTTATGAAAGTTTCTCTAACTTCCAATCTACACCAACAACAGAA 3831

CM70 3690 GCTGAATATGCTAATGTGCAAATTGCAAACAATACTGATTTATACAAGAGTTGGGGAATT 3749

||||||||||||||||||||||||||||||||||||||||||||||||||||||||||||

NRRL 3832 GCTGAATATGCTAATGTGCAAATTGCAAACAATACTGATTTATACAAGAGTTGGGGAATT 3891

CM70 3750 ACGAACTTCGAGTTTCCACCACAATATCGTTCAAGTACGGATAGTAGTTTCTTAGATTCA 3809

||||||||||||||||||||||||||||||||||||||||||||||||||||||||||||

NRRL 3892 ACGAACTTCGAGTTTCCACCACAATATCGTTCAAGTACGGATAGTAGTTTCTTAGATTCA 3951

CM70 3810 ATTATTCAAAATGGTTATGCATTTACTGATCGTTATGATCTTGGATTCAATACACCAACG 3869

||||||||||||||||||||||||||||||||||||||||||||||||||||||||||||

NRRL 3952 ATTATTCAAAATGGTTATGCATTTACTGATCGTTATGATCTTGGATTCAATACACCAACG 4011

CM70 3870 AAGTATGGTACTGTGGATCAACTCCGTACAGCTATTAAAGCTTTGCATGCGACAGGTATC 3929

|||||||||||||| |||||||||||||||||||||||||||||||||||||||||||||

NRRL 4012 AAGTATGGTACTGTAGATCAACTCCGTACAGCTATTAAAGCTTTGCATGCGACAGGTATC 4071

CM70 3930 AAGGCAATGGCAGATTGGGTACCAGATTTATAATTTGAAAGGTAAAGAAGTGGTTGCGGT 3989

||||||||||||||||||||||||||||||||||||||||||||||||||||||||||||

NRRL 4072 AAGGCAATGGCAGATTGGGTACCAGATTTATAATTTGAAAGGTAAAGAAGTGGTTGCGGT 4131

CM70 3990 ACAACGTGTCAACAACTCAGGAATCTATAATCAAGATTCTGTAATTAATAAAACATTATA 4049

||||||||||||||||||||||||||||||||||||||||||||||||||||||||||||

NRRL 4132 ACAACGTGTCAACAACTCAGGAATCTATAATCAAGATTCTGTAATTAATAAAACATTATA 4191

CM70 4050 TGCTTCACAAATCATTGGTGGCGGAGAATATCAGGCACTATATGGTGGAGAGTTCCTTGA 4109

||||||||||||||||||||||||||||||||||||||||||||||||||||||||||||

NRRL 4192 TGCTTCACAAATCATTGGTGGCGGAGAATATCAGGCACTATATGGTGGAGAGTTCCTTGA 4251

CM70 4110 TGAAATCAAGAAATTGTACCCTGCTCTATTCGAaaaaaaCCAAATTTCAACCGGCGTACC 4169

||||||||||||||||||||||||||||||||||||||||||||||||||||||||||||

NRRL 4252 TGAAATCAAGAAATTGTACCCTGCTCTATTCGAAAAAAACCAAATTTCAACCGGCGTACC 4311

CM70 4170 AATGGATGCTAGTGAAAAGATAAAAGAATGGTCCGCTAAGTACTTTAACGGTACTAACAT 4229

||||||||||||||||||||||||||||||||||||||||||||||||||||||||||||

NRRL 4312 AATGGATGCTAGTGAAAAGATAAAAGAATGGTCCGCTAAGTACTTTAACGGTACTAACAT 4371

CM70 4230 TCAAGGTCGTGGTGCTTACTATGTCCTTAAGGACTGGGCTACAAATGAGTACTTCAAGGT 4289

||||||||||||||||||||||||||||||||||||||||||||||||||||||||||||

NRRL 4372 TCAAGGTCGTGGTGCTTACTATGTCCTTAAGGACTGGGCTACAAATGAGTACTTCAAGGT 4431

CM70 4290 AAGCACATCAAGCAATAGCAATGTATTTTTGCCAAAGCAGTTGACGAATGAAGAATCAAA 4349

||||||||||||||||||||||||||||||||||||||||||||||||||||||||||||

NRRL 4432 AAGCACATCAAGCAATAGCAATGTATTTTTGCCAAAGCAGTTGACGAATGAAGAATCAAA 4491

CM70 4350 CACTGGATTTATTTCAACTGATGGTGGGATGACATATTATTCTACAAGTGGATACCAGGC 4409

||||||||||||||||||||||||||||||||||||||||||||||||||||||||||||

NRRL 4492 CACTGGATTTATTTCAACTGATGGTGGGATGACATATTATTCTACAAGTGGATACCAGGC 4551

CM70 4410 AAAAGATACATTCATCCAAGATGACAAATCTAATTGGTATTACTTTGACAAGAATGGTTA 4469

||||||||||||||||||||||||||||||||||||||||||||||||||||||||||||

NRRL 4552 AAAAGATACATTCATCCAAGATGACAAATCTAATTGGTATTACTTTGACAAGAATGGTTA 4611

CM70 4470 TATGACATATGGTTTCCAGACAGTCAATGATAATAATTATTACTTCTTGCCTAATGGTAT 4529

||||||||||||||||||||||||||||||||||||||||||||||||||||||||||||

NRRL 4612 TATGACATATGGTTTCCAGACAGTCAATGATAATAATTATTACTTCTTGCCTAATGGTAT 4671

CM70 4530 TGAATTACAAGATGCTATCTTAGAAGATAGTAAAGGAAATGTTTATTATTTCAATCAATA 4589

||||||||||||||||||||||||||||||||||||||||||||||||||||||||||||

NRRL 4672 TGAATTACAAGATGCTATCTTAGAAGATAGTAAAGGAAATGTTTATTATTTCAATCAATA 4731

CM70 4590 TGGCAAACAAGCTGTTGATGGATACTACATGTTGGCTAATAAAACTTGGCGTTACTTTGA 4649

||||||||||||||||||||||||||||||||||||||||||||||||||||||||||||

NRRL 4732 TGGCAAACAAGCTGTTGATGGATACTACATGTTGGCTAATAAAACTTGGCGTTACTTTGA 4791

CM70 4650 CAAAAATGGTGTTATGGCTAATGCTGGCTTAACAACCGTGACTGTTGATGGGCAGGTGCA 4709

||||||||||||||||||||||||||||||||||||||||||||||||||||||||||||

NRRL 4792 CAAAAATGGTGTTATGGCTAATGCTGGCTTAACAACCGTGACTGTTGATGGGCAGGTGCA 4851

CM70 4710 TATCCAATACTTTGATAAGAACGGTATTCAGGTCAAAGGGACTTCCGTGAAAGATGCAGA 4769

||||||||||||||||||||||||||||||||||||||||||||||||||||||||||||

NRRL 4852 TATCCAATACTTTGATAAGAACGGTATTCAGGTCAAAGGGACTTCCGTGAAAGATGCAGA 4911

CM70 4770 CGGAAAGCTACGCTACTTTGACCATGATTCTGGTGATATGGAGGCGACTCGCTTTGGTGA 4829

|||||||||||||||||||||| ||||||||||||||||| | ||| |||||||||||

NRRL 4912 CGGAAAGCTACGCTACTTTGACACTGATTCTGGTGATATGGTGACGAACCGCTTTGGTGA 4971

CM70 4830 AAACACAGATGGTTCATGGTCATACTTTGGTGCTGACGGTATCGCTGTATCTGGCGCTCA 4889

||||||||||||| ||||||||||||||||||||||||||||||||||| |||| || ||

NRRL 4972 AAACACAGATGGTACATGGTCATACTTTGGTGCTGACGGTATCGCTGTAACTGGTGCACA 5031

CM70 4890 GACAATTAGTGGGCAAAAATTGTTCTTTGATGCTGGCGGTCAACAGATTAAAGGTAAGGA 4949

||||||||||||||||||||||||||||||||||| ||| ||||||||||||||||||||

NRRL 5032 GACAATTAGTGGGCAAAAATTGTTCTTTGATGCTGACGGACAACAGATTAAAGGTAAGGA 5091

CM70 4950 AGCGTCTGATAAAAAGGGCAAAGTGCATTATTATGATGCTAATTCTGGTGAAATGATCGC 5009

|||| ||||||||||||||||||||||||||||||||||||||||||||||||||||| |

NRRL 5092 AGCGACTGATAAAAAGGGCAAAGTGCATTATTATGATGCTAATTCTGGTGAAATGATCAC 5151

CM70 5010 TAATCGTTTTGAAAAGTTATCAGATGGATCATGGGCGTACTTTAATAAAAAAGGTAACAT 5069

||||||||||||||||||||||||||||||||||||||||||||||||||||||||||||

NRRL 5152 TAATCGTTTTGAAAAGTTATCAGATGGATCATGGGCGTACTTTAATAAAAAAGGTAACAT 5211

CM70 5070 CGTAACCGGCGCACAAGTCATTAATGGTCAACATTTGTTCTTTGAAAGCAATGGTAACCA 5129

||||||||||||||||||||||||||||||||||||||||||||||||||||||||||||

NRRL 5212 CGTAACCGGCGCACAAGTCATTAATGGTCAACATTTGTTCTTTGAAAGCAATGGTAACCA 5271

CM70 5130 AGTTAAGGGTCGTGAATACACGGCTACTGATGGGAAGATGCGCTACTACGATGCAGATTC 5189

||||||||||||||||||||||||||||||||||||||||||||||||||||||||||||

NRRL 5272 AGTTAAGGGTCGTGAATACACGGCTACTGATGGGAAGATGCGCTACTACGATGCAGATTC 5331

CM70 5190 TGGTGATATGGTGACGAATCGCTTTGAACGAATATCAGACGGATCATGGGCATATTTTGA 5249

||||||||||||||||||||||||||||||||||||||||||||||||||||||||||||

NRRL 5332 TGGTGATATGGTGACGAATCGCTTTGAACGAATATCAGACGGATCATGGGCATATTTTGA 5391

CM70 5250 TGCTAATGGTGTTGCTGTATCTGGGGAACAAAATATAAATGGACAACAACTGTATTTTGA 5309

||||||||||||||||||| ||||||||||||||||||||||||||||||||||||||||

NRRL 5392 TGCTAATGGTGTTGCTGTAACTGGGGAACAAAATATAAATGGACAACAACTGTATTTTGA 5451

CM70 5310 TGCCAATGGTCATCAAGTTAAGGGAGCCGCAGTAAAACAAGCTGGCGGTAGCCAAAAATA 5369

|||||||||||||||||||||||||||||||||||||||||||| |||||||||||||||

NRRL 5452 TGCCAATGGTCATCAAGTTAAGGGAGCCGCAGTAAAACAAGCTGACGGTAGCCAAAAATA 5511

CM70 5370 TTATGACCCAAATTCTGGGGA 5390

||||||| |||||||||| ||

NRRL 5512 TTATGACGCAAATTCTGGAGA 5532

**Supplementary Figure S3.** Blast alignment of the *Leuconostoc mesenteroides* CM70 *dsrLL* gene (CM70, GenBank: MK401907) and *Lc. mesenteroides* NRRL B-512F *dsrT* gene (NRRL, GenBank: AB020020.1) as well as their corresponding upstream regions.


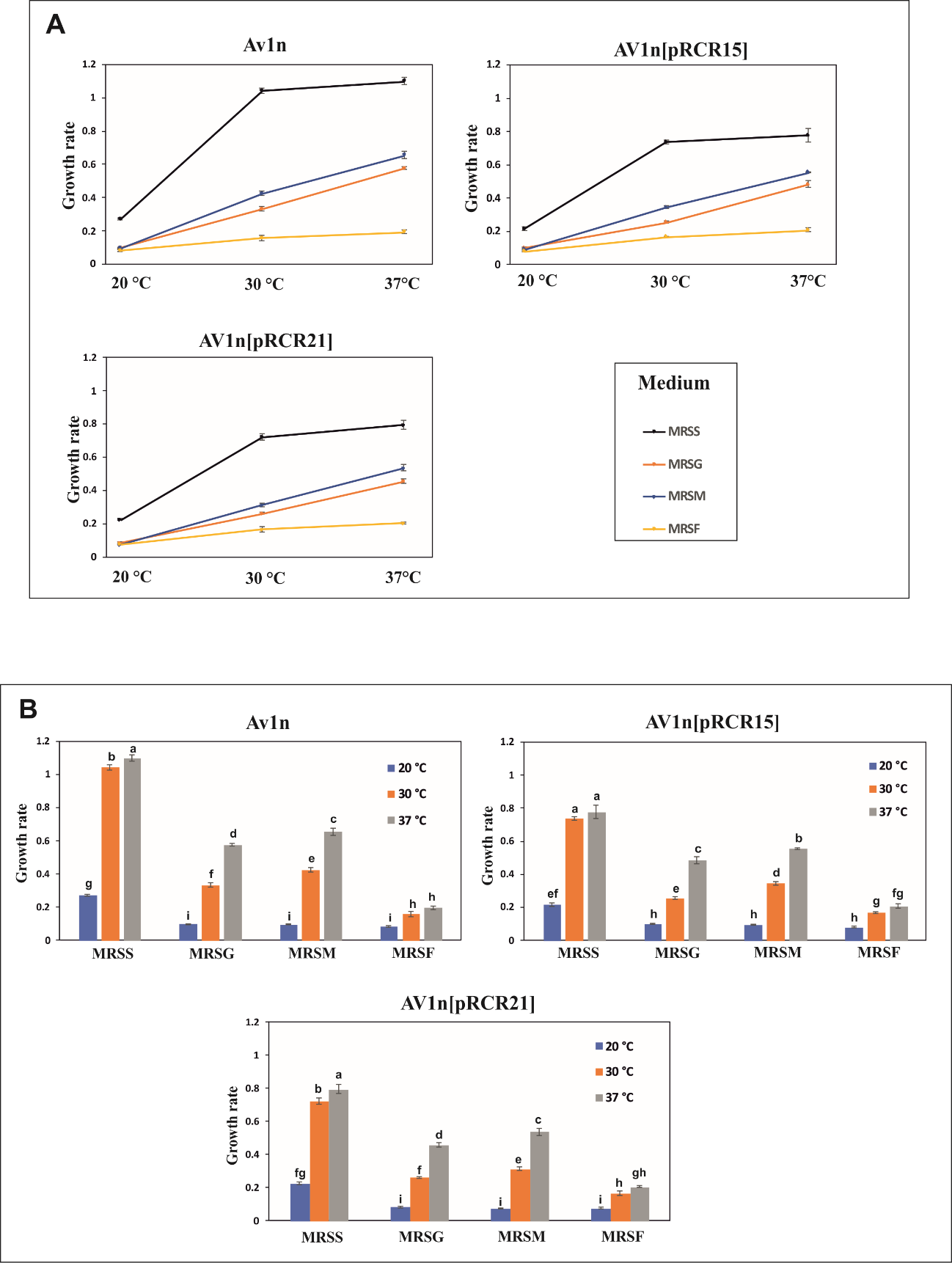


**Supplementary Figure S4.** Influence of temperature and carbon source on bacterial growth. The bacterial growth of the indicated strains was represented as a function of the growth temperature **(A)** or the growth medium **(B)**. Also, in **(B)** the ANOVA statistical analysis of the results is depicted. A *p* value [≤](https://fr.wiktionary.org/wiki/%E2%89%A4) 0.05 was considered significant. Mean pairwise comparisons were computed with a Tukey's test (α=0.05). Means with the same letter were not significantly different.


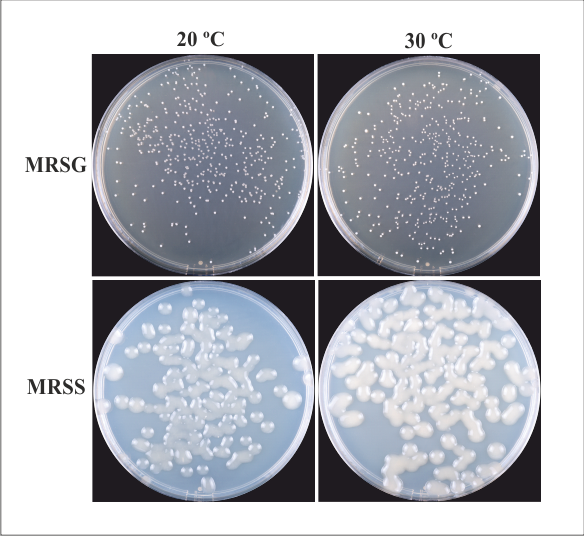


**Supplementary Figure S5**. EPS production by *Lactobacillus sakei* MN1 in solid media. Pictures of the plates were taken after 4 days of growth on MRS agar supplemented with 2% sucrose (MRSS) or 2% glucose (MRSG).

DsrLS TMW1411 1 MLRNNYFGETKTHYKLYKCGKNWAVMGISLFPLGLGMLVTSQPVSADVTATSTSSSAVRT 60

Consensus MLRNNYFGETKTHYKLYKCGKNWAVMGISLFPLGLGMLVTSQPVSADVTATSTSSSAVRT

DsrLS MN1 1 MLRNNYFGETKTHYKLYKCGKNWAVMGISLFPLGLGMLVTSQPVSADVTATSTSSSAVRT 60

DsrLS TMW1411 61 DAISESSSSAAKAETTSASSSSAVKAETTSASSSSAAKAETAAITTAGVANADSQTSAEV 120

Consensus DAISESSSSAAKAETTSASSSSAVKAETTSASSSSAAKAETAAITTAGVANADSQTSAEV

DsrLS MN1 61 DAISESSSSAAKAETTSASSSSAVKAETTSASSSSAAKAETAAITTAGVANADSQTSAEV 120

DsrLS TMW1411 121 TADSTSTSQVVTNNSNNQNNTAQPAGQEAAPVSEDTSSDDSERTTPTVANNDKPAIDSVD 180

Consensus TADSTSTSQVVTNNSNNQNNTAQPAGQEAAPVSEDTSSDDSERTTPTVANNDKPAIDSVD

DsrLS MN1 121 TADSTSTSQVVTNNSNNQNNTAQPAGQEAAPVSEDTSSDDSERTTPTVANNDKPAIDSVD 180

DsrLS TMW1411 181 TSQPATAAPKADTDVSTLQVDATTKTDSDIKEDTPTDKTTDTKTVQLTTVEGTSKQVVTT 240

Consensus TSQPATAAPKADTDVSTLQVDATTKTDSDIKEDTPTDKTTDTKTVQLTTVEGTSKQVVTT

DsrLS MN1 181 TSQPATAAPKADTDVSTLQVDATTKTDSDIKEDTPTDKTTDTKTVQLTTVEGTSKQVVTT 240

DsrLS TMW1411 241 PKEESSTDKSSSVVSKQTDKTSLPTVATATATTVSKIPSVTGDYQFDEKTKTYTFTGKDG 300

Consensus PKEESSTDKSSSVVSKQTDKTSLPTVATATATTVSKIPSVTGDYQFDEKTKTYTFTGKDG

DsrLS MN1 241 PKEESSTDKSSSVVSKQTDKTSLPTVATATATTVSKIPSVTGDYQFDEKTKTYTFTGKDG 300

DsrLS TMW1411 301 HPVTGLVYANNILQYFDETGHQVKGQYVTIAGHVYYFDPASGAAQTGVNQIDGKMVGFKS 360

Consensus HPVTGLVYANNILQYFDETGHQVKGQYVTIAGHVYYFDPASGAAQTGVNQIDGKMVGFKS

DsrLS MN1 301 HPVTGLVYANNILQYFDETGHQVKGQYVTIAGHVYYFDPASGAAQTGVNQIDGKMVGFKS 360

DsrLS TMW1411 361 DGSQITSGFSNDNAGNSYYFDESGTMVTGRQTIAGKTYYFDKDGHLRKGYSTIIDNQLYY 420

Consensus DGSQITSGFSNDNAGNSYYFDESGTMVTGRQTIAGKTYYFDKDGHLRKGYSTIIDNQLYY

DsrLS MN1 361 DGSQITSGFSNDNAGNSYYFDESGTMVTGRQTIAGKTYYFDKDGHLRKGYSTIIDNQLYY 420

DsrLS TMW1411 421 FDLKTGESVSTTTSNFKSGLTSQTDDTTPHNSAVNMSKDSFTTVDGFLTAESWYVPKDIQ 480

Consensus FDLKTGESVSTTTSNFKSGLTSQTDDTTPHNSAVNMSKDSFTTVDGFLTAESWYVPKDIQ

DsrLS MN1 421 FDLKTGESVSTTTSNFKSGLTSQTDDTTPHNSAVNMSKDSFTTVDGFLTAESWYVPKDIQ 480

DsrLS TMW1411 481 TSATDWRASTPEDFRPIMMTWWPTKQIQAAYLNHMVSEGLLSSDKKFSATDDQTLLNQAA 540

Consensus TSATDWRASTPEDFRPIMMTWWPTKQIQAAYLNHMVSEGLLSSDKKFSATDDQTLLNQAA

DsrLS MN1 481 TSATDWRASTPEDFRPIMMTWWPTKQIQAAYLNHMVSEGLLSSDKKFSATDDQTLLNQAA 540

DsrLS TMW1411 541 HAVQLQIELKIQQTKSVEWLRTTMHNFIKSQPGYNVTSETPSNDHLQGGALSYINSVLTP 600

Consensus HAVQLQIELKIQQTKSVEWLRTTMHNFIKSQPGYNVTSETPSNDHLQGGALSYINSVLTP

DsrLS MN1 541 HAVQLQIELKIQQTKSVEWLRTTMHNFIKSQPGYNVTSETPSNDHLQGGALSYINSVLTP 600

DsrLS TMW1411 601 DANSNFRLMNRNPTQQDGTRHYNTDTSEGGYELLLANDVDNSNPVVQAEQLNWLYFLTHF 660

Consensus DANSNFRLMNRNPTQQDGTRHYNTDTSEGGYELLLANDVDNSNPVVQAEQLNWLYFLTHF

DsrLS MN1 601 DANSNFRLMNRNPTQQDGTRHYNTDTSEGGYELLLANDVDNSNPVVQAEQLNWLYFLTHF 660

DsrLS TMW1411 661 GEIVKNDPSANFDSVRVDAVDNVDADLLNITAAYFRDVYGVDKNDLTANQHLSILEDWGH 720

Consensus GEIVKNDPSANFDSVRVDAVDNVDADLLNITAAYFRDVYGVDKNDLTANQHLSILEDWGH

DsrLS MN1 661 GEIVKNDPSANFDSVRVDAVDNVDADLLNITAAYFRDVYGVDKNDLTANQHLSILEDWGH 720

DsrLS TMW1411 721 NDPLYVKDHGSDQLTMDDYMHTQLIWSLTKNPDNRSAMRRFMEYYLVDRAKDNTSDQAIP 780

Consensus NDPLYVKDHGSDQLTMDDYMHTQLIWSLTKNPDNRSAMRRFMEYYLVDRAKDNTSDQAIP

DsrLS MN1 721 NDPLYVKDHGSDQLTMDDYMHTQLIWSLTKNPDNRSAMRRFMEYYLVDRAKDNTSDQAIP 780

DsrLS TMW1411 781 NYSFVRAHDSEVQTVIGDIVAKLYPDVKNSLAPSMEQLAAAFKVYDADMNSVNKKYTQYN 840

Consensus NYSFVRAHDSEVQTVIGDIVAKLYPDVKNSLAPSMEQLAAAFKVYDADMNSVNKKYTQYN

DsrLS MN1 781 NYSFVRAHDSEVQTVIGDIVAKLYPDVKNSLAPSMEQLAAAFKVYDADMNSVNKKYTQYN 840

DsrLS TMW1411 841 MPAAYAMLLTNKDTIPRVYYGDMYTDDGQYMATKSPYYDAISALLKARIKYVAGGQTMAV 900

Consensus MPAAYAMLLTNKDTIPRVYYGDMYTDDGQYMATKSPYYDAISALLKARIKYVAGGQTMAV

DsrLS MN1 841 MPAAYAMLLTNKDTIPRVYYGDMYTDDGQYMATKSPYYDAISALLKARIKYVAGGQTMAV 900

DsrLS TMW1411 901 DKHDILTSVRFGDGIMNASDKGSTTARTQGIGVIVSNNDALALKGDTVTLHMGIAHANQA 960

Consensus DKHDILTSVRFGDGIMNASDKGSTTARTQGIGVIVSNNDALALKGDTVTLHMGIAHANQA

DsrLS MN1 901 DKHDILTSVRFGDGIMNASDKGSTTARTQGIGVIVSNNDALALKGDTVTLHMGIAHANQA 960

DsrLS TMW1411 961 YRALLLTTTDGLMKYTSDNGAPIRYTDANGDLIFTSADIKGYQNVEVSGFLSVWVPVGAS 1020

Consensus YRALLLTTTDGLMKYTSDNGAPIRYTDANGDLIFTSADIKGYQNVEVSGFLSVWVPVGAS

DsrLS MN1 961 YRALLLTTTDGLMKYTSDNGAPIRYTDANGDLIFTSADIKGYQNVEVSGFLSVWVPVGAS 1020

DsrLS TMW1411 1021 DTQDARATGSSAANKTGDTLHSNAALDSNVIYEGFSNFQEMPTTHDEFTNVKIAQNADLF 1080

Consensus DTQDARATGSSAANKTGDTLHSNAALDSNVIYEGFSNFQEMPTTHDEFTNVKIAQNADLF

DsrLS MN1 1021 DTQDARATGSSAANKTGDTLHSNAALDSNVIYEGFSNFQEMPTTHDEFTNVKIAQNADLF 1080

DsrLS TMW1411 1081 KSWGVTSFQLAPQYRSSDDTSFLDSIIKNGYAFTDRYDLGFNTPTKYGDVDDLADAIRAM 1140

Consensus KSWGVTSFQLAPQYRSSDDTSFLDSIIKNGYAFTDRYDLGFNTPTKYGDVDDLADAIRAM

DsrLS MN1 1081 KSWGVTSFQLAPQYRSSDDTSFLDSIIKNGYAFTDRYDLGFNTPTKYGDVDDLADAIRAM 1140

DsrLS TMW1411 1141 HSVGIQVMADFVPDQIYNLPGQEVVAVNRTNNFGTPNQDSDLQNQLYVTNSKGGGEYQAK 1200

Consensus HSVGIQVMADFVPDQIYNLPGQEVVAVNRTNNFGTPNQDSDLQNQLYVTNSKGGGEYQAK

DsrLS MN1 1141 HSVGIQVMADFVPDQIYNLPGQEVVAVNRTNNFGTPNQDSDLQNQLYVTNSKGGGEYQAK 1200

DsrLS TMW1411 1201 YGGEFLDLLRLEHPDLFTTNQISTGVPIDGSTKIKEWSAKYFNGSDIQGKGADYVLKDGA 1260

Consensus YGGEFLDLLRLEHPDLFTTNQISTGVPIDGSTKIKEWSAKYFNGSDIQGKGADYVLKDGA

DsrLS MN1 1201 YGGEFLDLLRLEHPDLFTTNQISTGVPIDGSTKIKEWSAKYFNGSDIQGKGADYVLKDGA 1260

DsrLS TMW1411 1261 SQEYFKITSNANDESFLPKQFMNQDAMTGFTTDEKGTTYYSTSGYQAKQSFIQGDDGQYY 1320

Consensus SQEYFKITSNANDESFLPKQFMNQDAMTGFTTDEKGTTYYSTSGYQAKQSFIQGDDGQYY

DsrLS MN1 1261 SQEYFKITSNANDESFLPKQFMNQDAMTGFTTDEKGTTYYSTSGYQAKQSFIQGDDGQYY 1320

DsrLS TMW1411 1321 YFDADGYMVTGSQTINGKQYYFLPNGVELREAFLQNASGNTVYYGKTGSAVKSKYVVDQS 1380

Consensus YFDADGYMVTGSQTINGKQYYFLPNGVELREAFLQNASGNTVYYGKTGSAVKSKYVVDQS

DsrLS MN1 1321 YFDADGYMVTGSQTINGKQYYFLPNGVELREAFLQNASGNTVYYGKTGSAVKSKYVVDQS 1380

DsrLS TMW1411 1381 GVAYYFDVNGNMVADRMMILDGHTQYFFAGGSQAKDQFLIGSDGNLRYFDQGSGNMVTNR 1440

Consensus GVAYYFDVNGNMVADRMMILDGHTQYFFAGGSQAKDQFLIGSDGNLRYFDQGSGNMVTNR

DsrLS MN1 1381 GVAYYFDVNGNMVADRMMILDGHTQYFFAGGSQAKDQFLIGSDGNLRYFDQGSGNMVTNR 1440

DsrLS TMW1411 1441 FAVNRNGDWFYFNGDGIALKGWQTIAGKTYFFDADGRQV-----KAAAEQAAAEQAAADK 1495

Consensus FAVNRNGDWFYFNGDGIALKGWQTIAGKTYFFDADGRQV KAAA++AAAEQAAADK

DsrLS MN1 1441 FAVNRNGDWFYFNGDGIALKGWQTIAGKTYFFDADGRQVKAAADKAAADKAAAEQAAADK 1500

DsrLS TMW1411 1496 AAADKAAAEQAAADKAAADKAAAEQAAADKAAADKAAAEQAAAEQAAADK-----AAAEQ 1550

Consensus AAADKAAAEQAAADKAAADKAAAEQAAADKAAADKAAAEQAAA++AAADK AAAEQ

DsrLS MN1 1501 AAADKAAAEQAAADKAAADKAAAEQAAADKAAADKAAAEQAAADKAAADKAAAEQAAAEQ 1560

DsrLS TMW1411 1551 AAADKAAAEQAAADKAAAEQAATDKAAADKAAAEQAAAEQAAAEQAAADKAAAEQAAADK 1610

Consensus AAADKAAAEQAAADKAAAEQAATDKAAADKAAAEQAAA++AAA++AAA++AAAEQAAADK

DsrLS MN1 1561 AAADKAAAEQAAADKAAAEQAATDKAAADKAAAEQAAADKAAADKAAAEQAAAEQAAADK 1620

DsrLS TMW1411 1611 AAAEQAAADKAAAEQAAADKAAADKAAAEQAAADKAAADKAAAEQAAAEQAAADKAAAEQ 1670

Consensus AAAEQAAADKAAAEQ AAADKAAAEQAAADKA

DsrLS MN1 1621 AAAEQAAADKAAAEQ-----AAADKAAAEQAAADKA------------------------ 1651

DsrLS TMW1411 1671 AAADKAAAEQAAAEQAAADKAAAKDKQTQAVAYAATKAKNNIDQATTADGINDAQATGIT 1730

Consensus AAKDKQTQAVAYAATKAKNNIDQATTADGINDAQATGIT

DsrLS MN1 1652 ---------------------AAKDKQTQAVAYAATKAKNNIDQATTADGINDAQATGIT 1690

DsrLS TMW1411 1731 DIDNQHVPGTSVDNQKQAEKVTEDIKNDPDNKTLPEAIELPNTGVDKTESITITGVVMLI 1790

Consensus DIDNQHVPGTSVDNQKQAEKVTEDIKNDPDNKTLPEAIELPNTGVDKTESITITGVVMLI

DsrLS MN1 1691 DIDNQHVPGTSVDNQKQAEKVTEDIKNDPDNKTLPEAIELPNTGVDKTESITITGVVMLI 1750

DsrLS TMW1411 1791 LTTIFGLLFTSKKHKKD 1807

Consensus LTTIFGLLFTSKKHKKD

DsrLS MN1 1751 LTTIFGLLFTSKKHKKD 1767

**Supplementary Figure S6.** Blast alignment of the *Lactobacillus sakei* TMW1.411 (translation from sequence 28 of WGS project published in DDBJ/ENA/GenBank under the accession QOSE00000000) and *Lb. sakei* MN1 (translated from GenBank ATN28243) dextransucrases amino acid sequences.
